# M2 Macrophage‐Mimetic and ROS‐Sensitive Hybrid Nanoplatform for Targeted Alleviation of Rheumatoid Arthritis

**DOI:** 10.1002/EXP.20230030

**Published:** 2026-08-19

**Authors:** Yi Xiao, Shiyu Meng, Zhuang Tang, Yonghang Liu, Xican Zhang, Kefan Wu, Yuanhao Liu, Ao Li, Jingyao Feng, Kaixiang Zhou, Hui Guo, Xiaolin Wang

**Affiliations:** ^1^ School of Pharmacy and State Key Laboratory of Quality Research in Chinese Medicine Macau University of Science and Technology Macao China; ^2^ Center For Advanced Materials Research Advanced Institute of Natural Sciences Beijing Normal University At Zhuhai Zhuhai China; ^3^ School of Chemical Engineering and Technology Sun Yat‐sen University Zhuhai China

**Keywords:** M2 macrophage‐mimetic, macrophage repolarization, ROS‐responsive, rheumatoid arthritis, targeted therapy

## Abstract

Rheumatoid arthritis (RA) constitutes the most prevalent inflammatory arthropathy globally, which is typically marked by synovitis and deteriorating joint damage. Regrettably, RA remains incurable owing to insufficient therapeutic response along with severe side reactions of available treatments. To overcome these formidable obstacles, an M2 macrophage membrane‐cloaked, ROS‐sensitive lipid‐gated, and herbal anti‐arthritic agent celastrol (Cel)‐loaded hybrid silica nanosystem (Cel@M2‐L‐MSN) was established to reshape the intricate RA microenvironment. Benefiting from cell membrane camouflaging, Cel@M2‐L‐MSN inherited the inflammation‐homing capability of M2 macrophages and selectively accumulated in the inflamed joints, where excessive ROS triggered Cel release from the mesopores to exert anti‐inflammatory and antioxidative effects for joint protection. Moreover, Cel@M2‐L‐MSN demonstrated strengthened anti‐inflammatory capability by robustly sponging multiple pro‐arthritogenic factors including TNF‐α, IL‐1β, and CCL‐2. In adjuvant‐induced arthritis (AIA) rats, Cel@M2‐L‐MSN tremendously alleviated arthritis symptoms, mitigated cartilage and bone degeneration, and attenuated major organ injuries by playing multiple roles including inflammation downregulation, ROS clearance, and macrophage repolarization. Furthermore, Cel@M2‐L‐MSN substantially enhanced the cyto/hemocompatibility of free Cel and manifested good biosafety in vivo. Collectively, this study presents a biohybrid and stimuli‐responsive nanomedicine for targeted RA remission, which also holds great promise for the clinical intervention of a wide spectrum of refractory inflammatory diseases.

## Graphical abstract

Rheumatoid arthritis (RA) is the most prevalent inflammatory arthropathy globally and remains incurable nowadays. Herein, an M2 macrophage‐mimetic hybrid nanoplatform (Cel@M2‐L‐MSN) is constructed for concerted RA therapy through joint‐specific accumulation, on‐demand drug release, inflammation resolution, ROS clearance, along with macrophage repolarization.

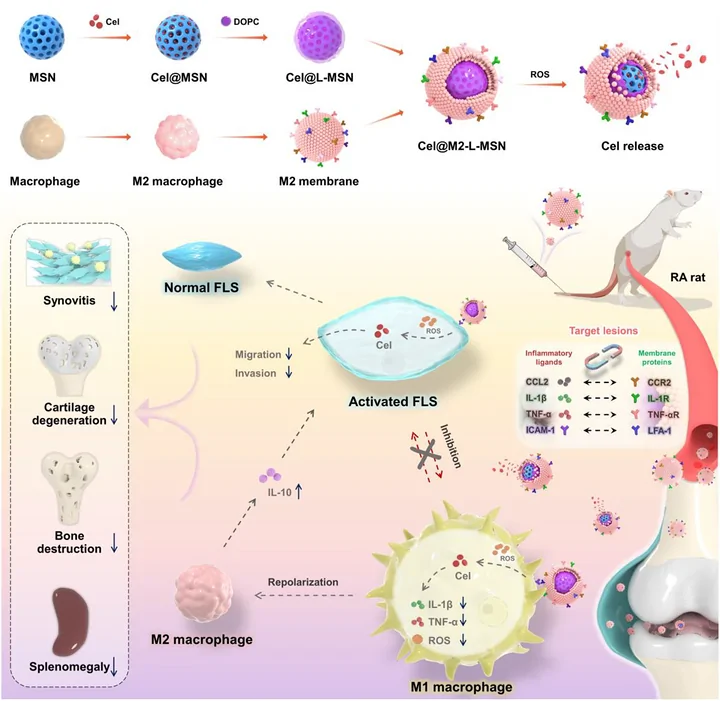

## 1 Introduction

Rheumatoid arthritis (RA) is a debilitating autoimmune disorder that involves nearly 1% of the population worldwide [1]. The synovial inflammation and progressive joint damage eventually lead to irreversible deformity, disability, and even mortality in RA patients. To make it worse, uncontrolled RA more often than not is accompanied by an array of comorbidities, including pulmonary interstitial fibrosis, cardiovascular diseases, and osteoporosis [2]. Although the pathogenesis of RA is poorly understood, risk factors including sex, aging, genetics, and immune dysfunctions have been reported to be responsible for the pathogenesis and increased prevalence of RA [1, 3]. To date, tremendous efforts have been dedicated to elucidating the etiology and developing therapeutic regimens for the devastating inflammatory disorder [4, 5].

The RA microenvironment is hallmarked by immune cell infiltration and synovial hyperplasia, which orchestrate pannus formation, articular cartilage destruction, and bone erosion [6]. The infiltrated immune cells produce pro‐inflammatory cytokines and stimulate fibroblast‐like synoviocytes (FLS) to exert pro‐inflammatory and tissue‐destructive effects by aggressive migration and invasion and excessive production of pathogenic mediators, including chemokines, reactive oxygen species (ROS), metalloproteinases, and receptor activator of nuclear factor kappa‐B (NF‐κB) ligands [7, 8]. Meanwhile, macrophages in RA synovium are highly plastic and can be polarized into two major phenotypes, namely, M1 and M2 macrophages [9, 10]. More specifically, M1 macrophages secrete proinflammatory cytokines (e.g., TNF‐α, IL‐1β, and IL‐6) to perpetuate inflammation, exacerbate joint destruction, and inhibit chondrogenesis, while M2 macrophages produce anti‐inflammatory cytokines, including IL‐10, to favor inflammation resolution and tissue regeneration. Therefore, joint‐specific pathogenic molecules and cells are potential therapeutic targets for efficient RA management.

Despite the fast advances in drug development, there remains no cure for RA in clinics [2, 11]. The first‐line drugs for RA intervention, including disease‐modifying antirheumatic drugs, glucocorticoids, nonsteroidal anti‐inflammatory drugs, and biologics, are frequently reported with poor therapeutic responses and side effects such as infections and tuberculosis during long‐term administration [4]. Recently, a large body of evidence has demonstrated that Chinese herb‐derived agents hold great potential as novel antirheumatic drugs [12]. Specifically, Celastrol (Cel) is a bioactive agent derived from the herb *Tripterygium wilfordii* that has been applied for the treatment of a variety of diseases such as RA, systemic lupus erythematosus, and cancer [13]. Most importantly, Cel is among the most promising candidates for clinical RA management and targets numerous signaling pathways involving NF‐κB, ROS, DNA damage, and apoptosis to suppress inflammation and protect cartilage and bone from degeneration [14]. Yet, the clinical application of Cel is hindered by low solubility and systemic cytotoxicity, emphasizing the urgent need for developing innovative drug delivery carriers [15, 16, 17].

Over the past decades, a variety of nanosystems have been designed and constructed for efficient RA intervention [18, 19, 20]. Among them, mesoporous silica nanoparticles (MSN) are extensively investigated drug carriers due to the ease of fabrication, tunable size and structure, high drug loading capacity, and biodegradability, which have been widely applied for the treatment of RA [21], bone regeneration [22], infection [23], cancers [24], and so on. In particular, silicon‐containing MSNs are bioactive and beneficial for biomineralization in bone and cartilage engineering [19, 25]. Moreover, gatekeepers (e.g., polymer and ultrasmall nanoparticles) capping the mesopores of MSNs allow well‐controlled anti‐arthritic drug release under specific stimuli such as ROS [26], light [27], and ultrasound [28]. Furthermore, novel therapeutic strategies including targeted delivery [29], theranostic therapy [21], cell‐free DNA eradication [30], and enhanced tissue penetration [31] can be conceived to combat devastating RA by advanced particle functionalization. Cell membrane coating is an emerging surface‐engineering technology that potentiates the development of cell‐mimetic nanoformulations by transferring the cell membranes onto the surface of nanocores [32, 33]. Resultantly, the as‐established nanosystems are endowed with a series of bioactivities, including prolonged blood circulation, inflammation homing, and tumor targeting [34, 35]. To date, cell membranes deprived from mesenchymal stem cells [36], FLS [37], leukocytes [38, 39], platelets [40], or their combination with erythrocytes [41, 42] have been reported to generate cell‐mimetic nanosystems targeting RA alleviation. Therefore, cell‐membrane‐camouflaged silica nanoplatforms hold great promise for RA therapy by harnessing the pharmaceutical merits of the MSN nanocore and the biological functions of the cell membrane coating.

**SCHEME 1:**
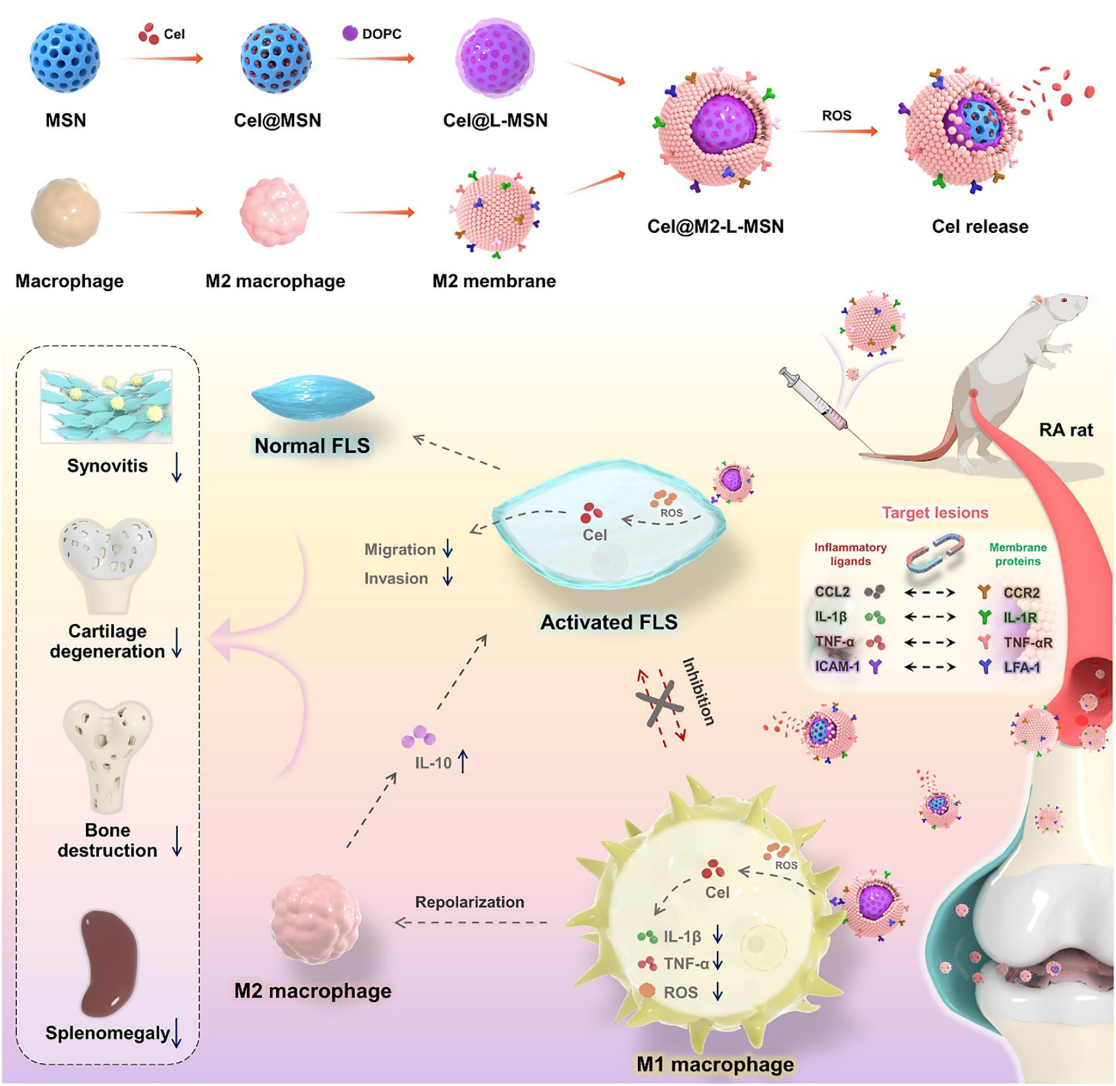
Construction of M2 macrophage‐mimetic hybrid nanoplatform (Cel@M2‐L‐MSN) for concerted RA therapy through joint‐specific accumulation, on‐demand drug release, inflammation resolution, and ROS clearance, along with macrophage repolarization.

Based on the complicated RA microenvironment and the inflammation‐resolving property of M2 macrophages, we herein report on an M2 macrophage membrane‐cloaked hybrid mesoporous silica nanoplatform (Cel@M2‐L‐MSN) for efficient RA therapy. As illustrated in Scheme 1, Cel‐loaded MSN was sealed with a ROS‐sensitive lipid layer and further engineered with the M2 macrophage membrane. The as‐obtained M2 macrophage‐mimetic nanoplatform was characterized by high drug loading, ROS‐responsive drug release, substantially enhanced cyto/hemocompatibility, and selective internalization by inflammatory FLS and macrophages, inflammatory cyto/chemokine absorption, ROS scavenging, inhibition of FLS invasiveness, and macrophage reprogramming capability in vitro. Furthermore, the comprehensive histological analyses and micro‐computed tomography validated that Cel@M2‐L‐MSN successfully reduced synovial hyperplasia, attenuated cartilage and bone degradation, downregulated inflammation, and mitigated multiple organ damage in AIA rats. Taken together, Cel@M2‐L‐MSN served as a multifunctional nanomedicine to surmount the complexity of RA.

## 2 Results and Discussions

### 2.1 Preparation and Characterization of Cel@M2‐L‐MSN

**FIGURE 1:**
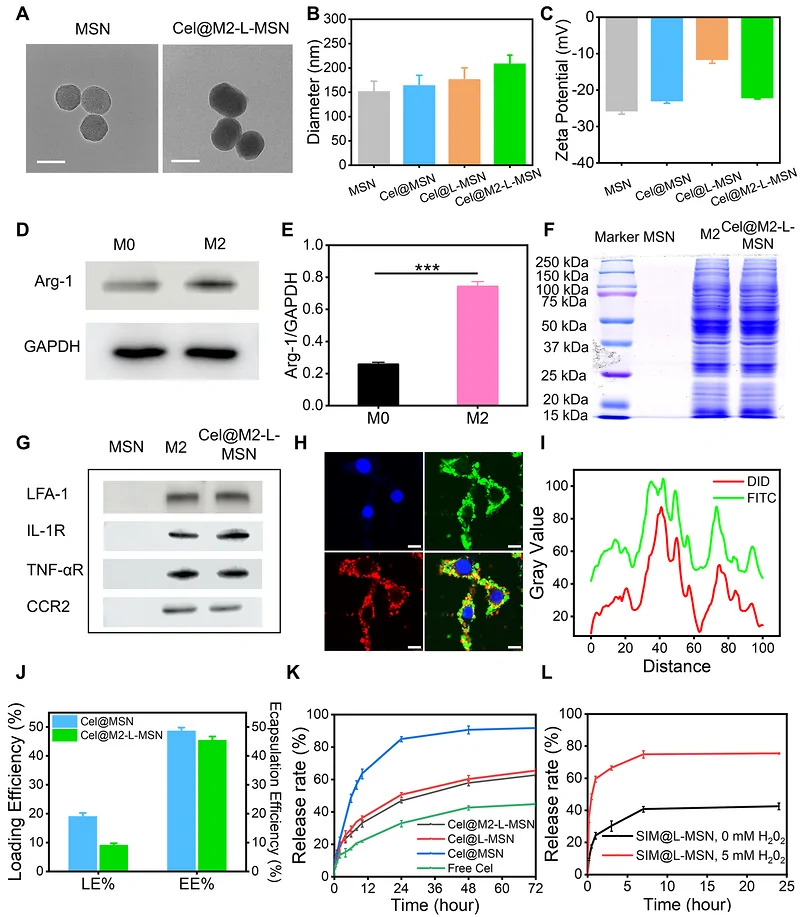
Characterizations of Cel@M2‐L‐MSN. (A) Morphology of MSN and Cel@M2‐L‐MSN observed by TEM (scale bar = 100 nm). (B) Particle sizes and (C) Zeta potentials of MSN, Cel@L‐MSN, Cel@M2‐L‐MSN, and M2. (D) Expression of Arg‐1 and Image J (E) Protein composition of MSN, M2, and Cel@M2‐L‐MSN as disclosed by (F) Coomassie blue staining and (G) Western blotting assayS. Stability of Cel@M2‐L‐MSN following internalization by LPS‐activated RAW 264.7 cells at 37°C for 4 h was validated by (H) CLSM observations (green for FITC, red for DiD, and blue for DAPI; scale bar = 10 µm) and (I) Co‐localization analysis by ImageJ. (J) The loading efficiency (LE%) and encapsulation efficiency (EE%) of Cel@MSN and Cel@M2‐L‐MSN. (K) Drug release behavior of Free Cel (green), Cel@MSN (blue), Cel@L‐MSN (red), and Cel@M2‐L‐MSN (black) at 37°C in PBS (pH = 7.4). (L) The drug release profile of SIM@L‐MSN in the presence or absence of 5 mM H_2_O_2_. Data were expressed as mean ± SD with *n* = 3.

The preparation route of the hybrid nanosystem (Cel@M2‐L‐MSN) was illustrated in Scheme 1. After MSNs fabrication and Cel loading, the mesopores of the as‐obtained nanoparticles (Cel@MSN) were capped with a 1,2‐dioleoyl‐sn‐glycero‐3‐phosphocholine (DOPC)‐containing lipid shell (Cel@L‐MSN) and further camouflaged by M2 macrophage cell membrane by sonication (Cel@M2‐L‐MSN). As revealed by transmission electron microscopy (TEM) observation, bare MSN was spherical with uniform size and well‐defined mesoporous structures, which is beneficial for drug loading (Figure 1A). In comparison, the masked mesopores and newly formed outer layer of Cel@M2‐L‐MSN indicated successful assembly of the hybrid nanosystem. Moreover, DLS measurement disclosed that the hydrodynamic diameter gradually increased from 151 ± 20 nm for bare MSN to 208 ± 18 nm for Cel@M2‐L‐MSN upon drug loading and hybrid membrane coating (Figure 1B). In addition, the zeta potential of MSN was elevated from −25 mV to −11 mV after drug and lipid introduction, which was reversed to −22 mV upon M2 macrophage membrane cloaking, suggesting recovered colloidal stability of Cel@M2‐L‐MSN (Figure 1C).

The mesoporous characteristics of MSN were validated by Brunauer‒Emmett‒Teller (BET) analysis of nitrogen adsorption‐desorption isotherms, which demonstrated a classical type IV isotherm (Figure S1). Besides, the specific surface area, pore volume, and pore size were 1097.51 m^2^/g, 1.499 cm^3^/g, and 2.8 nm, respectively. As expected, the mesopores of Cel@L‐MSN were obscured, and the surface area and pore volume were sharply reduced to only 5.85% and 3.35% of bare MSN, respectively, indicating successful mesopore capping. Moreover, MSN exhibited three characteristic peaks at 1226 cm^−1^, 1050 cm^−1^, and 802 cm^−1^ in the infrared spectrum, which corresponded to the asymmetric and symmetric stretching vibration of Si‐O‐Si (Figure S2). Interestingly, new peaks attributed to the vibration of carbonyl groups (C = O) appeared at 1728 cm^−1^ and 1589 cm^−1^ in the spectrum of Cel@MSN, revealing successful Cel loading into MSN. Furthermore, the X‐ray diffraction (XRD) pattern of Cel powder exhibited multiple sharp peaks at 2θ values of 9.5, 14.9, 17, and 18.9 (Figure S3). However, the characteristic peaks associated with Cel disappeared in the diffraction pattern of Cel@MSN. Hence, Cel existed in the amorphous state after MSN encapsulation.

Recently, M2 rather than M1 macrophage membrane‐coated nanoplatforms have exhibited higher efficacy for inflammatory disorder treatment due to superior inflammation‐sponging capability [41, 43, 44] Hence, RAW 264.7 cells were polarized into M2 macrophages before membrane deprivation, which was characterized by significantly upregulated Arg‐1 expression (Figures 1D and E) [43]. Sodium dodecyl sulfate‐polyacrylamide gel electrophoresis (SDS‐PAGE) results disclosed identical protein band patterns for pure M2 vesicles and Cel@M2‐L‐MSN, indicating successful translocation of the M2 membrane onto the silica nanosurface (Figure 1F). Besides, the western blotting assay validated the presence of crucial membrane proteins such as lymphocyte function‐associated antigen 1 (LFA‐1), TNF‐α receptor (TNF‐αR), IL‐1β receptor (IL‐1R), and C–C motif chemokine receptor 2 (CCR2), which were assumed to respectively bind with overexpressed ICAM‐1, TNF‐α, IL‐1β, and CCL2 in the milieu of the arthritic joint (Figure 1G) [45]. Further, the integrity of Cel@M2‐L‐MSN after cellular internalization by inflammatory RAW 264.7 cells was validated by confocal laser scanning microscopy (CLSM) with M2 and MSNs labeled with DiD (red) and fluorescein isothiocyanate (FITC, green), respectively (Figure 1H). In the merged fluorescent image, the strong yellow signals resulting from the overlapping of red and green fluorescence demonstrated the successful formation and excellent stability of M2 macrophage membrane‐coated nanosystems. Moreover, co‐localization analysis by ImageJ further confirmed the successful M2 macrophage membrane camouflaging over the nanocore (Figure 1I).

The drug loading and drug release profile of Cel@M2‐L‐MSN was determined by UV‐vis spectrophotometry. As can be seen in Figure S4, Cel exhibited a maximum absorption wavelength at 425nm, where the nanoparticles showed no interference. Therefore, the UV‐vis spectrophotometry method developed in this study demonstrated excellent specificity for quantitative analysis of Cel. Despite further surface coating and purification, Cel was loaded into Cel@M2‐L‐MSN at a high loading efficiency of 9.0±0.5% as determined by exploiting the absorbance at 425 nm (Figure 1J). Regarding the drug release kinetics, free Cel demonstrated retarded and incomplete release (< 45% over 72 h) due to poor solubility and dissolution (green line, Figure 1K). On the contrary, the drug release rate and amount of Cel@MSN were substantially enhanced, which exceeded 90% at 72 h (blue line). Interestingly, the drug release of nanoparticles was retarded after lipid capping, and the cumulative Cel release was ca. 60% in 72 h for both Cel@L‐MSN (red line) and Cel@M2‐L‐MSN (black line). In terms of ROS‐responsive drug release behaviors, Cel‐loaded nanomedicine generally adopts stable hydrophobic molecules as substitutes due to the chemical instability of Cel in hydrogen peroxide (H_2_O_2_), the main component of ROS [46, 47]. Thereafter, simvastatin (SIM) was alternatively encapsulated into L‐MSN to monitor the ROS‐triggered drug release of Cel@M2‐L‐MSN. As shown in Figure 1L, the release rate and cumulative release amount of SIM were much higher upon exposure to 5 mM H_2_O_2_ than in pure PBS, indicating a marked ROS‐sensitive drug release capability resulting from the lipid layer breakdown in oxidative conditions [48]. These data indicated that Cel@M2‐L‐MSN can potentially achieve Cel enrichment in the RA joint by preferential drug release in the oxidative microenvironment compared with normal tissues.

### 2.2 Cellular Internalization

To explore the targetability in vitro, the cellular internalizations of the M2 macrophage‐mimetic nanoplatforms were monitored using FITC as a fluorescence indicator. Besides, MH7A (human rheumatoid fibroblast‐like synoviocytes) and RAW 264.7 cells were activated by lipopolysaccharide (LPS) and were adopted as inflammatory fibroblast‐like synoviocytes (FLS) and macrophage models, respectively, both of which were verified by enhanced expression of intercellular cell adhesion molecule‐1 (ICAM‐1) (Figures S5–S6). Meanwhile, non‐stimulated cells were employed as controls.

**FIGURE 2:**
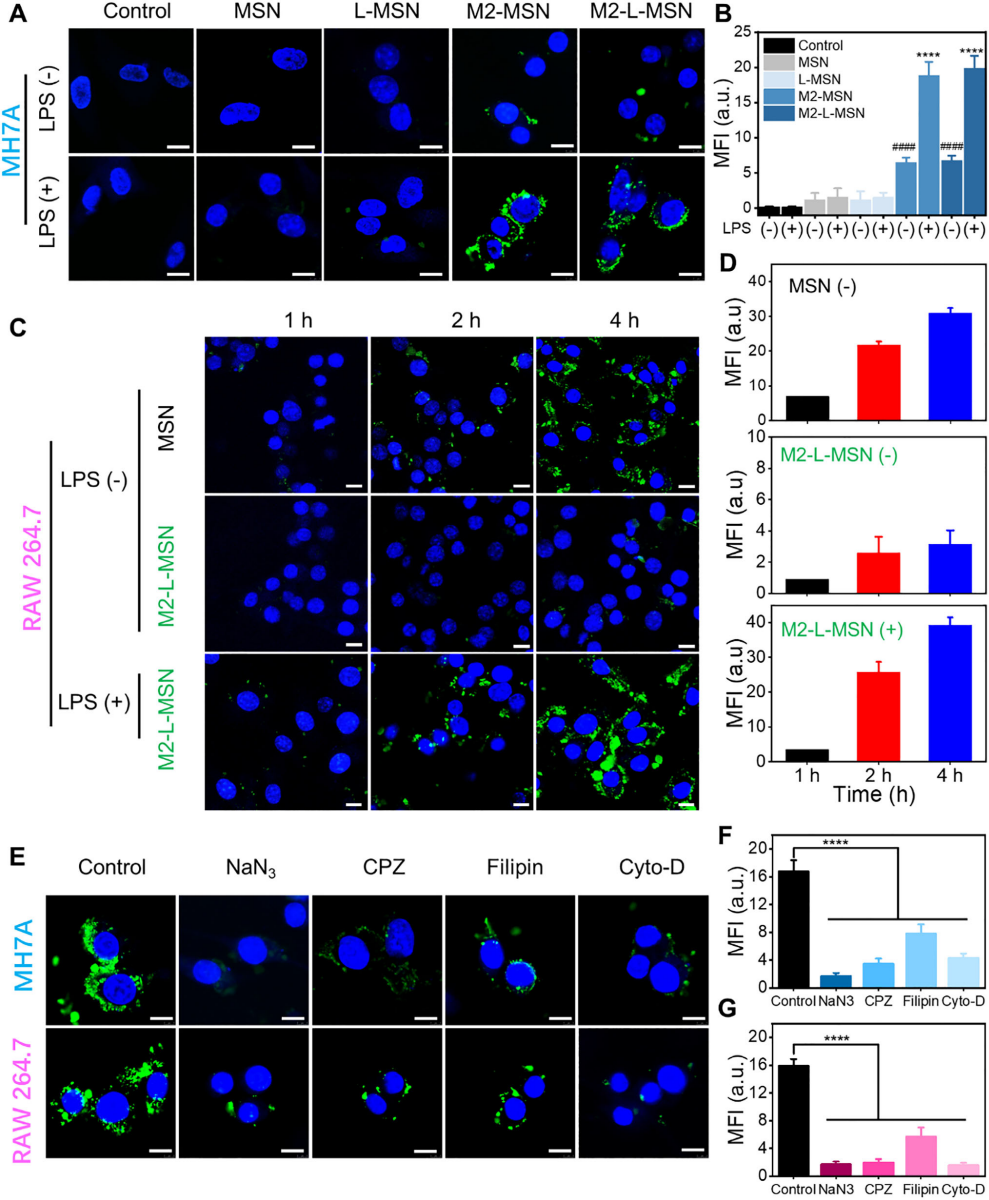
Cellular uptake behaviors of M2 macrophage‐mimetic nanoparticles in MH7A and RAW 264.7 cells. (A) Intracellular localization of M2‐L‐MSN‐FITC (green) in MH7A cells in the presence (+) or absence (−) of LPS stimulation and (B) corresponding quantification of mean fluorescence intensity (MFI) by ImageJ (*indicates the difference among groups with LPS activation while # represents the variance among groups without LPS activation). (C) Cellular uptake of MSN‐FITC (green) and M2‐L‐MSN‐FITC (green) in RAW264.7 cells in the presence (+) or absence (−) of LPS stimulation, and (D) corresponding MFI quantification of internalized FITC‐labeled nanoparticles with [M2‐L‐MSN (+)] or without [MSN (−), M2‐L‐MSN (−)] LPS activations at 1 h, 2 h, and 4 h. (E) Endocytosis of M2‐L‐MSN‐FITC by LPS‐activated MH7A and RAW264.7 cells in the presence of varying inhibitors, and (F) corresponding quantitative analysis of MFI by ImageJ. The nucleus was stained with DAPI (blue). Scale bar, 10 µm. Data were expressed as mean ± SD with *n* = 3. ^∗∗∗∗^ *p* < 0.0001, and ^####^ *p* < 0.0001.

Without cell membrane coating, MSN‐FITC and L‐MSN‐FITC demonstrated low green fluorescent signals in MH7A cells regardless of LPS activation, suggesting deficient and non‐differential cellular internalization (Figures 2A and B) [49]. In contrast, the fluorescence intensity of M2‐MSN‐FITC and M2‐L‐MSN‐FITC was dramatically higher in LPS‐activated MH7A cells, which was around 2.9‐fold that of non‐LPS‐stimulated counterparts. Furthermore, the cellular uptake efficiency of M2‐MSN‐FITC in MH7A cells was comparable with M2‐L‐MSN‐FITC, indicating that the inner lipid layer didn't impair the internalization efficiency of the biomimetic nanosystems. These data validated that M2‐L‐MSN‐FITC demonstrated preferential internalization by inflammatory FLS, which was predominantly enhanced by the M2 membrane camouflaging.

Afterward, the uptake efficiencies of the biomimetic nanoformulations were evaluated in normal or LPS‐induced RAW264.7 cells after 1–4 h incubation. As shown in Figures 2C and D, the gradually intensified fluorescent signals suggested time‐dependent cell internalization for all tested groups. Strikingly, MSN‐FITC demonstrated strong fluorescence intensity, whereas barely any fluorescence was observed in the M2‐L‐MSN‐FITC group after co‐incubation with normal macrophages for 4 h, indicating that M2 macrophage camouflaging furnished an effective strategy to escape from macrophage phagocytosis [45]. In sharp contrast, dramatically enhanced internalization was observed for M2‐L‐MSN‐FITC incubated in LPS‐activated RAW264.7 cells. The above findings demonstrated that M2‐L‐MSN‐FITC can not only prolong circulation time by escaping clearance by normal macrophages but also facilitate nanoparticle accumulation in RA joints by actively targeting inflammatory macrophages and FLS.

Furthermore, the endocytosis mechanism of M2‐L‐MSN in LPS‐activated MH7A and RAW264.7 cells was examined in the presence of various inhibitors [38]. Interestingly, the cellular uptake of M2‐L‐MSN in inflammatory MH7A cells witnessed a tremendous reduction (53.38% ‐ 89.86%) by the inhibitors NaN_3_, CPZ, Filipin, and Cyto‐D, which disrupt ATP production, clathrin‐mediated endocytosis, caveolae‐mediated endocytosis, and actin dynamics, respectively (Figures 2E‐F). Meanwhile, the cellular internalization of M2‐L‐MSN in LPS‐activated RAW264.7 cells shared the same trend as that of MH7A cells, the reduction of which ranged from 63.93% to 89.93% in comparison with the control group (Figures 2E, G). As a whole, the M2‐L‐MSN internalization in inflammatory MH7A or RAW264.7 cells was energy‐dependent and mediated by multiple endocytosis pathways [38, 50].

### 2.3 Therapeutic Potentials in Vitro

**FIGURE 3:**
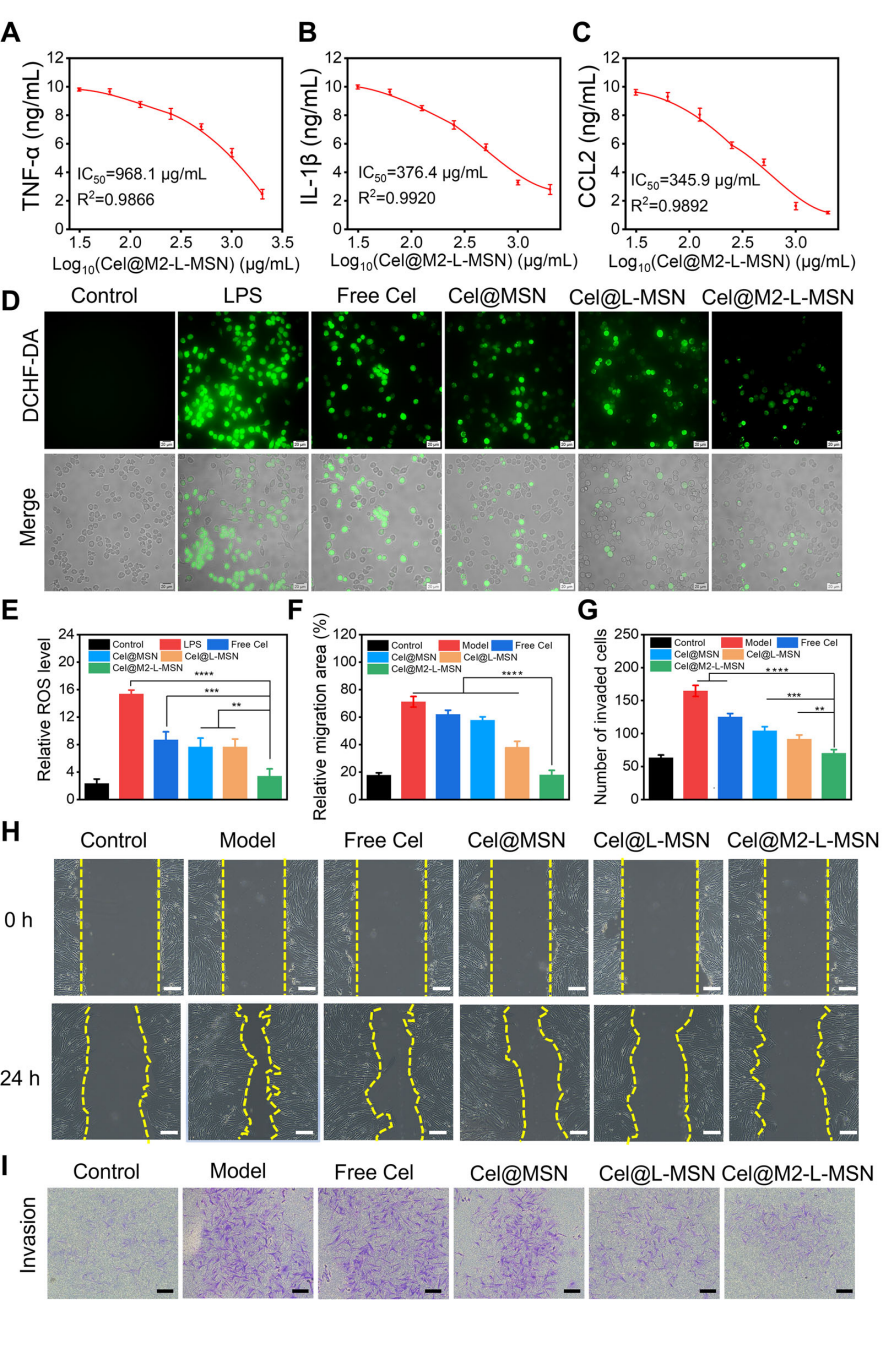
The therapeutic potential of Cel@M2‐L‐MSN in vitro. Binding kinetics of inflammatory factors including (A) TNF‐α, (B) IL‐1β, and (C) CCL2. (D) Fluorescent images and (E) relative intracellular ROS levels in RAW264.7 cells of various groups (scale bar = 20 µm). Quantification analysis of (F) relative migration area of MH7A cells and (G) number of invaded cells resulted from (H) wound scratch of monolayer of MH7A cells and (I) Transwell‐mediated invasion experiments, respectively (scale bar = 200 µm). Data were expressed as mean ± SD with *n* = 3. ^∗∗^ *p* < 0.01, ^∗∗∗^ *p* < 0.001, and ^∗∗∗∗^ *p* < 0.0001.

Biological therapies targeting inflammatory cytokines and chemokines are effective in RA intervention, which plays detrimental roles in RA development and pathogenesis [8, 11]. Thus, the sponging potentials of Cel@M2‐L‐MSN to inflammatory cytokines (TNF‐α and IL‐1β) and chemokines (CCL2) were determined by ELISA tests. As shown in Figures 3A–C, Cel@M2‐L‐MSN robustly absorbed the inflammatory factors in a concentration‐dependent manner. The calculated IC_50_ (half maximal inhibitory concentration) of TNF‐α, IL‐1β, and CCL2 was 968.1 µg/mL, 376.4 µg/mL, and 345.9 µg/mL, respectively, following Hill's equation, indicating strong inflammatory cytokine and chemokine clearance ability [51].

ROS accumulation contributes to the initiation and maintenance of inflammation and tissue damage in RA [52, 53]. The ROS scavenging ability of Cel@M2‐L‐MSN was monitored by adopting DCFH‐DA as the ROS probe in LPS‐activated RAW264.7 cells. The dramatically elevated intracellular ROS levels by LPS stimulation resulted in significantly strengthened fluorescent intensity, which was 6.6‐fold that of the control group (Figure 3D). In comparison, Cel@M2‐L‐MSN treatment demonstrated high ROS scavenging efficiency and sharply eliminated the ROS level to 22.1%, 39.3%, 44.6%, and 43.7% that of the model, Cel, Cel@MSN, and Cel@L‐MSN groups, respectively (Figure 3E).

Moreover, wound scratch and transwell‐mediated invasion tests were performed to assess the inhibitory effects of Cel@M2‐L‐MSN on the migration and invasion of activated FLS (Figures 3F–I). The results showed that the migration rate of LPS‐stimulated MH7A cells was dramatically enhanced compared with that in the control group, which was suppressed after various treatments (Figures 3F and H). Notably, the relative migration area of the Cel@M2‐L‐MSN group was as low as one‐fourth that of the model group, which was similar to that of non‐activated cells. Furthermore, the Matrigel‐coated transwell was adopted to evaluate the invasiveness of FLS after Cel@M2‐L‐MSN treatment (Figures 3G and I). In parallel, LPS stimulation increased the invasive MH7A cell numbers to 2.6‐fold that of the control group, while Cel@M2‐L‐MSN treatment dramatically decreased invasive cell numbers, which were 42.5%, 55.8%, 67.1%, and 76.4% that of the model, free Cel, Cel@MSN, and Cel@L‐MSN groups, respectively.

To conclude, Cel@M2‐L‐MSN demonstrated multiple anti‐RA effects, including robust neutralization of inflammatory cytokines (TNF‐α and IL‐1β) and chemokines (CCL2), efficient scavenging of intracellular ROS, as well as significant suppression of FLS migration and invasion. The concentration‐dependent absorption profiles and IC_50_ values confirmed the potent inflammatory factor clearance capacity of this biomimetic nanoplatform. Meanwhile, its superior ROS elimination ability highlighted its potential to alleviate oxidative stress‐driven synovial inflammation and joint erosion. Furthermore, the marked inhibition of FLS aggressiveness indicated that Cel@M2‐L‐MSN could potentially restrain pannus formation and cartilage destruction. Collectively, these multifunctional therapeutic actions positioned Cel@M2‐L‐MSN as a promising and comprehensive strategy for RA intervention, meriting further in vivo investigation.

### 2.4 Macrophage Repolarization in Vitro

**FIGURE 4:**
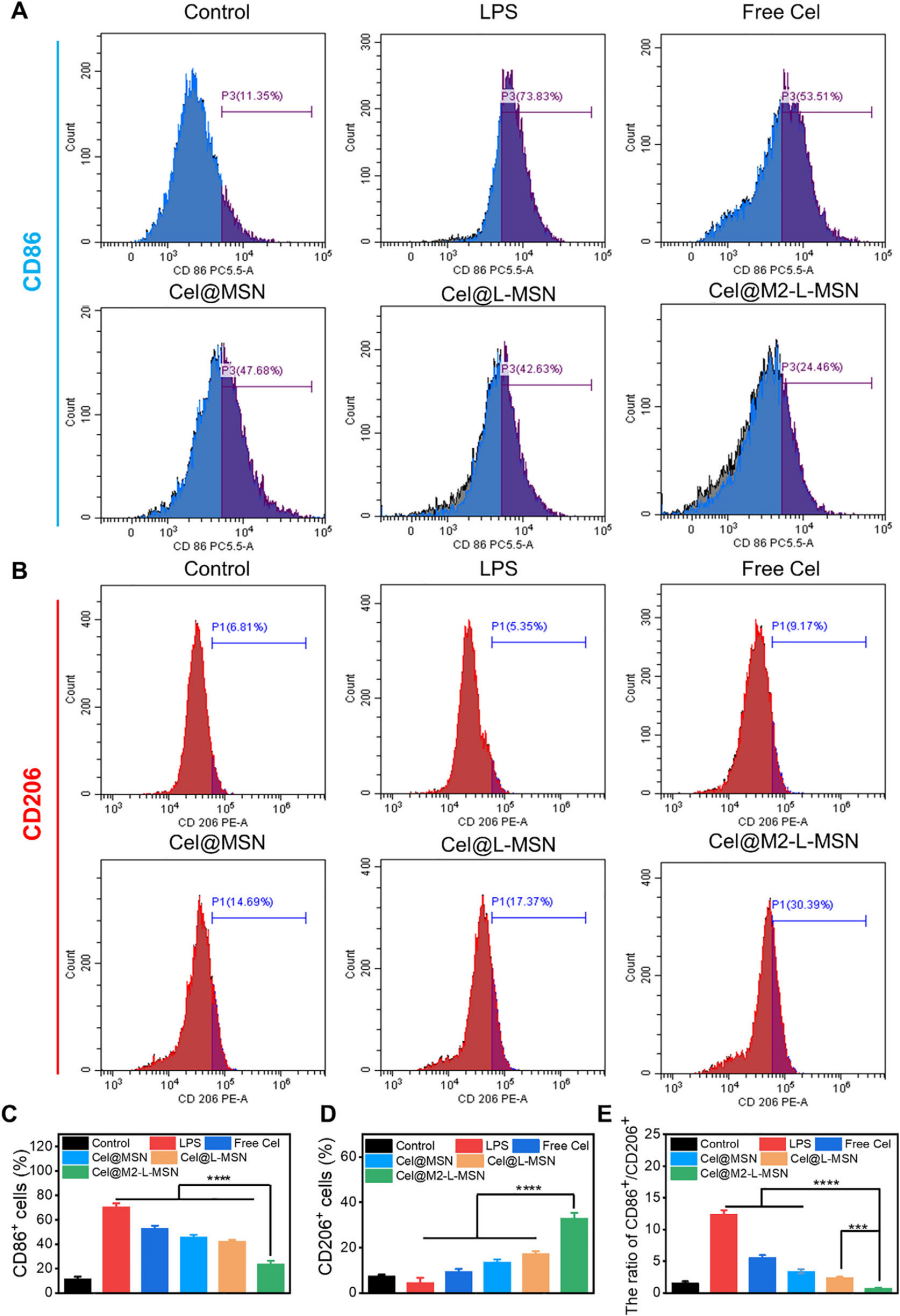
Macrophage repolarization effects of Cel@M2‐L‐MSN in vitro. Flow cytometry histograms of (A) M1 macrophages and (B) M2 macrophages labeled by CD86 and CD206, respectively, from different groups. The quantitative analysis of (C) CD86‐positive (+) and (D) CD206‐positive (+) cell percentages. (E) The ratio of CD86+/CD206+ was calculated from flow cytometry analysis. Data were expressed as mean ± SD for *n* = 3. ^∗∗∗^ *p* < 0.001, ^∗∗∗∗^ *p* < 0.0001.

Mounting evidence shows that predominant M1 macrophage polarization in the joints aggravates RA progression [9, 10]. Therefore, reprogramming the phenotypic transition of macrophages is a promising approach for RA therapy. In our study, the macrophages were immunofluorescence stained with CD86 (M1 marker) and CD206 (M2 marker), and the effect of Cel@M2‐L‐MSN on macrophage repolarization was evaluated by flow cytometry. Consistent with previous reports, LPS activation markedly increased the proportion of M1 macrophages to 73.83%, indicating a typical M1 phenotype transition (Figures 4A–E) [21, 54]. On the contrary, Cel nanoformulations reversed the phenotypic transition to various degrees. Notably, Cel@M2‐L‐MSN substantially decreased the M1/M2 ratio from 12.4 in the model group to as low as 0.7, thereby demonstrating the most prominent effect in inducing M1 to M2 repolarization.

The production of inflammatory cytokines is significantly upregulated in RA joints, leading to edema, stiffness, pain, and tissue destruction [6]. Therefore, the expressions of TNF‐α and IL‐1β produced by LPS‐activated RAW 264.7 cells were analyzed by ELISA tests to further investigate the anti‐inflammatory capability of Cel@M2‐L‐MSN (Figures S7A–B). As a result, both free Cel and Cel nanoformulations significantly attenuated the TNF‐α and IL‐1β expressions, among which Cel@M2‐L‐MSN exhibited the optimal inhibitory effects. It was speculated that intracellular ROS accelerated Cel release from Cel@M2‐L‐MSN, which scavenged ROS, successfully transformed macrophages from M1 into M2 phenotype, and substantially attenuated pro‐inflammatory cytokine production. Collectively, we verified the M1 to M2 repolarization of macrophages by Cel@M2‐L‐MSN that synergistically scavenges ROS and sponges inflammatory mediators.

### 2.5 Cytotoxicity and Hemocompatibility Assay

The cytotoxicity of various formulations was assayed on MH7A and RAW 264.7 cells [55, 56]. The drug‐free nanocarriers, namely, MSN, L‐MSN, and M2‐L‐MSN, demonstrated desirable cytocompatibility at 20–400 µg/mL (cell viability > 90%), suggesting good safety of the vehicle material for clinical applications (Figure S8). Moreover, free Cel exhibited high cytotoxicity in these two cell lines, which was consistent with previous reports (Figure S9‐10) [17, 57, 58]. Nonetheless, there was no cytotoxicity of Cel@M2‐L‐MSN at the equivalent drug concentration (Cel concentrations ranging from 50 to 800 ng/mL), which could be attributed to the fact that free Cel in the molecular forms can freely penetrate the cells while Cel@M2‐L‐MSN exhibited a controlled drug release profile (Figures 1K–L) and low cellular uptake efficiency in non‐activated MH7A and RAW 264.7 cells (Figures 2A–D). Our findings indicated that Cel@M2‐L‐MSN can efficiently eliminate the cytotoxicity of free Cel by on‐demand drug release and targeted delivery [57].

In terms of hemocompatibility, Milli‐Q water (positive control) and PBS (negative control) were employed as controls for all the measurements, the hemolysis rate of which was 100% and 0%, respectively, after incubation with red blood cells (RBC) for 3 h (Figure S11). Free Cel was reported to be hemolytic, and the hemolysis rate was as high as 15.8% at 50 µg/mL [59]. In our study, the hemolysis rate of the MSN and Cel@MSN groups exceeded 10% at 200 µg/mL and 100 µg/mL, respectively, which continued to increase to ca. 35 % at 600 µg/mL (Figures S11A–B). Therefore, incorporation in the mesopores of MSN alone was insufficient to reduce the high hemolysis risk of Cel. Conversely, Cel@M2‐L‐MSN witnessed minimal hemolysis (< 3%) at the range of 20–600 µg/mL (equivalent to 2.84‐85.2 µg/mL Cel), indicating substantially enhanced safety for intravenous injection (Figures S11C–D). Therefore, Cel@M2‐L‐MSN demonstrated excellent cyto/hemocompatibility for further investigations in vivo.

### 2.6 Pharmacokinetics and Biodistribution Studies

**FIGURE 5:**
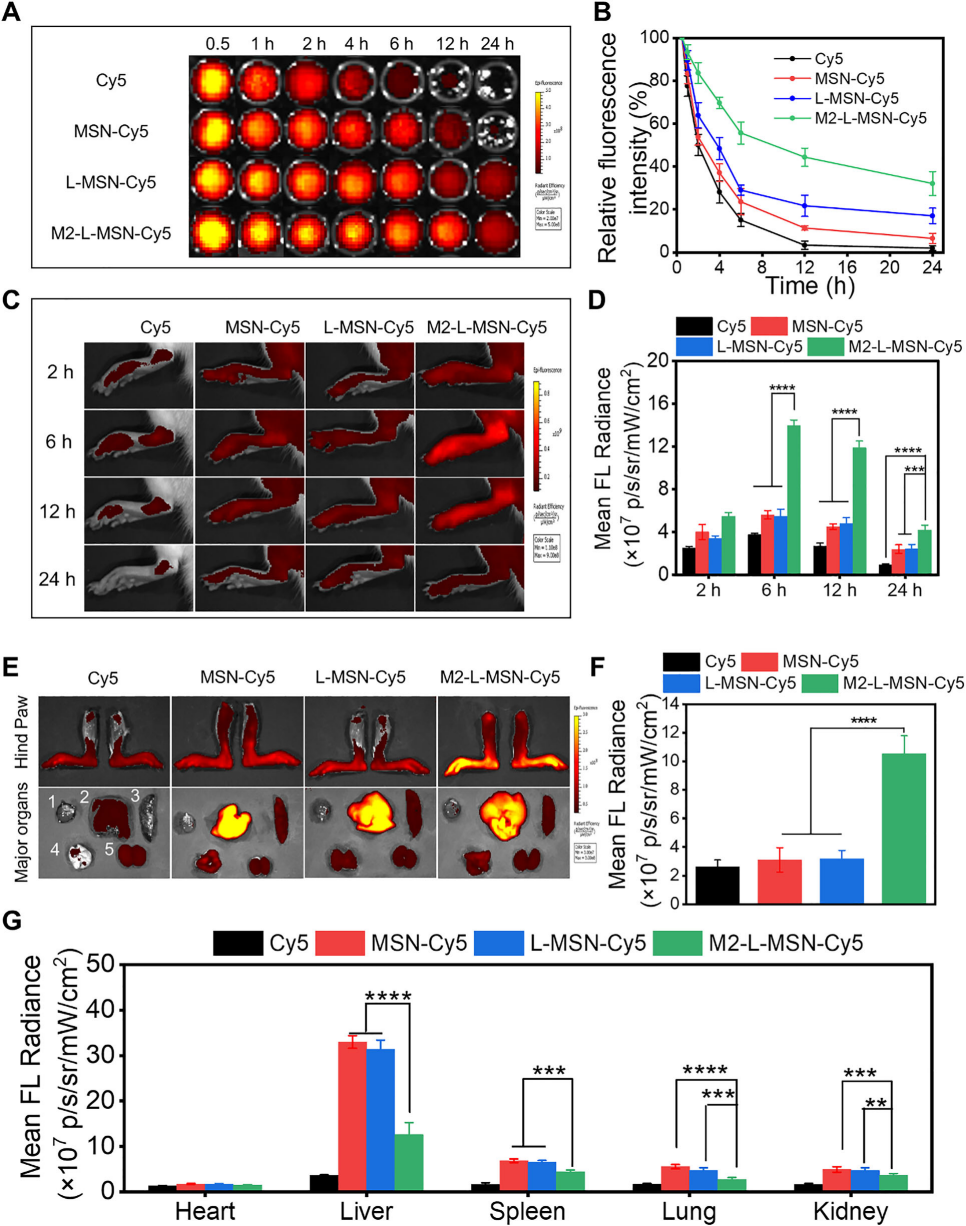
Pharmacokinetics and biodistribution of M2‐L‐MSN in AIA rats. (A) Fluorescence imaging and (B) relative fluorescence intensity of whole blood from AIA rats after intravenous injections. (C) Accumulation of Cy5 or Cy5‐labeled formulations in the hind paws from different groups in vivo and (D) corresponding mean fluorescence radiance analysis of hind paws at various time points. (E) *Ex vivo* accumulation of Cy5 or Cy5‐labeled nanoparticles in the hind paws and main organs (1. Heart, 2. Liver, 3. Spleen, 4. Lungs, and 5. Kidneys) 24 h after injections. MFI quantifications of the (F) hind paws and (G) major organs. Data were expressed as mean ± SD with *n* = 3. ^∗∗^ *p* < 0.01, ^∗∗∗^ *p* < 0.001, and ^∗∗∗∗^ *p* < 0.0001.

The prolonged circulation and targeted biodistribution of nanoparticles can boost the therapeutic outcomes and circumvent the systemic toxicity of drugs in the management of RA [60]. To investigate the pharmacokinetics of M2 macrophage mimic nanosystems, adjuvant‐induced arthritis (AIA) rats were injected with Cy5 or Cy5‐labeled formulations via the tail vein, and the fluorescence intensity of the whole blood was measured at predetermined time intervals. As shown in Figures 5A and B, the fluorescence intensity of all the groups was the highest immediately upon administration and gradually trailed off over time. MSN prolonged the circulation time of Cy5, which was further improved after lipid capping due to the presence of PEG. Benefiting from the immune‐evasion property of the M2 macrophage camouflaging, the M2‐L‐MSN‐Cy5 group exhibited the slowest decline rate in blood circulation [61].

Moreover, the live animal fluorescence imaging disclosed that the hind paws of AIA rats in the Cy5 and L‐MSN‐Cy5 groups exhibited relatively weak fluorescent signals from 2 h to 24 h post‐injection due to insufficient enrichment (Figures 5C and D). In contrast, M2‐L‐MSN‐Cy5 demonstrated dramatically strengthened fluorescence signals in the RA joints at all predetermined time points. Notably, the fluorescent intensity of M2‐L‐MSN‐Cy5 in the hind paws was 2.53‐fold and 2.48‐fold that of the L‐MSN‐Cy5 and MSN‐Cy5 groups 6 h after injection, indicating selective accumulation in inflamed joints. Subsequently, the hind paws, together with the major organs of the rats, were harvested 24 h after various treatments, and the corresponding fluorescence intensity was measured *ex vivo*. Consistent with the in vivo data, M2 membrane coating dramatically improved the accumulation of M2‐L‐MSN‐Cy5 at the RA joints compared with other groups (Figures 5E,F). Furthermore, nanoparticles without appropriate surface functionalizations preferentially accumulate in the metabolic organs due to elimination by the reticuloendothelial system (RES) [55]. Nonetheless, the enrichment of M2‐L‐MSN‐Cy5 in the livers and spleens was reduced to ca. 40% and 65% of that of MSN and L‐MSN‐Cy5, respectively owing to the immune evasive properties imparted by M2 macrophage membrane engineering (Figure 5E,G). In addition, the non‐specific distribution of M2‐L‐MSN was also reduced in other organs such as the lung and kidney.

Based on the data of cellular uptake observations (Figures 2A–D), cyto‐/ chemokine binding tests (Figures 3A–C), biodistribution studies (Figures 5C–G), and previous reports [45], it can be inferred that multiple factors, including the upregulation of ICAM‐1 in inflammatory synoviocytes and macrophages, along with the overexpression of inflammatory mediators (e.g., TNF‐α, IL‐1β, and CCL2), were crucial for the selective distribution of M2‐L‐MSN to the inflamed joints. Remarkably, M2‐L‐MSN‐Cy5 exhibited potent joint targeting ability and long circulation time in AIA rats, which constituted an ideal nanocarrier in the targeted treatment of RA. Furthermore, it can be hypothesized that targeted delivery mediated by M2 macrophage membrane coating, combined with ROS‐responsive drug release facilitated by DOPC capping, ensures that CeL release from CeL@M2‐L‐MSN is specifically triggered by ROS in the inflammatory and oxidative RA joint. This targeted approach could potentially reduce the risk of adverse effects of Cel while maximizing its therapeutic efficacy.

### 2.7 Anti‐Arthritic Effects of Cel@M2‐L‐MSN In Vivo

In order to investigate the therapeutic outcomes of Cel@M2‐L‐MSN in the treatment of RA, AIA rats with an inflammation score higher than 4 were randomly grouped and intravenously injected with free Cel, Cel@MSN, Cel@L‐MSN, and Cel@M2‐L‐MSN, respectively, 12 days post‐modeling every other day for 16 days. Meanwhile, healthy and AIA rats (inflammation score ≥ 4) subjected to intravenous injection of PBS were utilized as the control and the model group, respectively.

**FIGURE 6:**
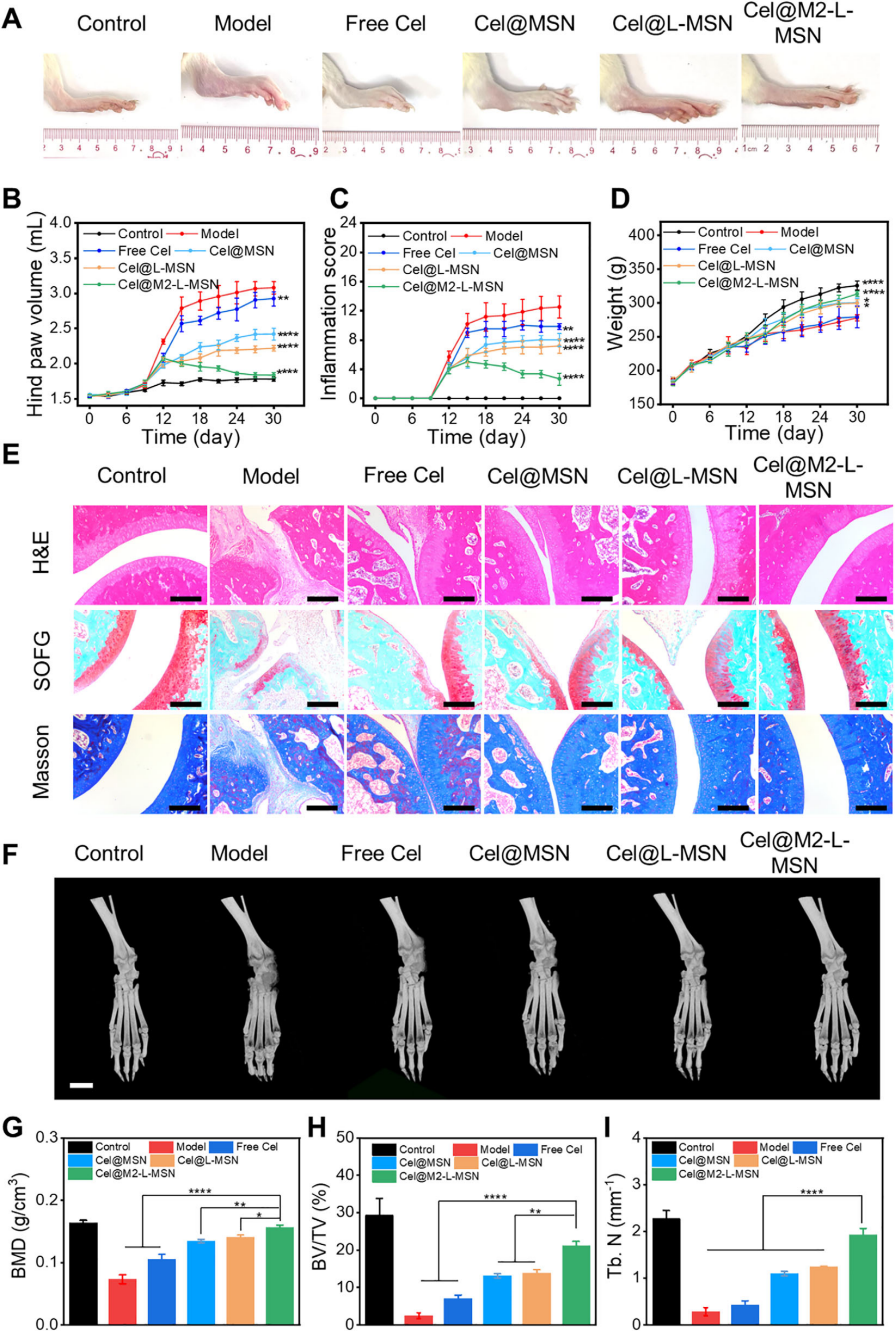
Therapeutic efficacy of Cel@M2‐L‐MSN in AIA rats. (A) Typical photographs of hind paws on day 30. (B) Hind paw volume, (C) inflammation score, (D) body weight of rats from different groups over one‐month treatment. (E) Histological analysis of the ankle joints after different treatments (scale bar = 100 µm). (F) Typical micro‐CT scanning images of the hind paws (scale bar = 5 mm) together with the corresponding quantification of (G) BMD, (H) BV/TV, and (I) Tb. N of different groups. Data were expressed as mean ± SD with *n* = 6. ^∗^ *p* < 0.05, ^∗∗^ *p* < 0.01, ^∗∗∗∗^ *p* < 0.0001.

The model group demonstrated redness and edema in the joints, while treatments with free Cel and nanoformulations ameliorated joint edema to varying degrees on day 30 (Figure 6A). Besides, the inflammation score and paw volume, along with body weights of all the groups, were measured every 3 days to monitor the RA progression (Figure 6B). Results showed that the inflammation score of the model group steadily increased to 12.5 ± 1.5 on day 30. After treatments with Cel formulations, the inflammation scores of AIA rats in the free Cel, Cel@MSN, Cel@L‐MSN, and Cel@M2‐L‐MSN groups decreased to 78.6%, 64.0%, 57.3%, and 21.3% of that of the model group, respectively. In consistency, the hind paw volumes after treatments with free Cel, Cel@MSN, Cel@L‐MSN, and Cel@M2‐L‐MSN were reduced to 94.3%, 78.0%, 71.5%, and 59.1% of that of the model group on day 30, respectively (Figure 6C). Inspiringly, the step‐by‐step reduction (Cel < Cel@MSN < Cel@L‐MSN < Cel@M2‐L‐MSN) in joint edema, inflammation score, and hind paw volume emphasized the necessity and importance of lipid capping and M2 membrane decoration for efficient RA relief.

Moreover, RA progression is associated with disturbed body growth [42]. Remarkably, the weight loss of AIA rats was mitigated following various treatments, among which the Cel@M2‐L‐MSN group recovered body weight to 96.2% of the healthy rats (Figure 6D). Furthermore, the inhibitory effects of Cel formulations on splenomegaly were studied by calculating the individualized body weight‐corrected spleen weights (spleen index) [62]. The spleens in the model group were abnormally enlarged, as depicted in Figure S12A. In addition, the spleen index of AIA rats exhibited a significant increase by 2.1‐fold in comparison to that of the control group, whereas administration of Cel@M2‐L‐MSN resulted in a substantial decrease in the spleen index to 60.6% of that of the model group, indicating robust splenomegaly attenuation (Figure S12B).

Furthermore, histological analyses of the ankle joints were conducted to study the articular pathophysiology after various treatments (Figure 6E). Hematoxylin‐eosin (H&E) staining of the model group manifested aggressive synovial hyperplasia and inflammatory cell infiltration together with reduced chondrocytes [63]. In contrast, the Cel@M2‐L‐MSN group demonstrated a broadened articular cavity, minimal inflammatory infiltration, and recovered cartilage integrity, verifying optimal therapeutic effects. Besides, cartilage erosion was further evaluated by the quantity of proteoglycan, which undergoes degradation during RA progression [51]. Saffranine O‐fast green (SOFG) staining in the model group demonstrated a significant decrease in the area and intensity of red staining for proteoglycan [64], which was normalized by Cel@M2‐L‐MSN due to successful preservation of cartilage. Moreover, collagen deposition in the articular cartilage matrix was revealed by Masson staining [65]. There was a substantial depletion of collagen in AIA rats, as evidenced by reduced staining area and density. On the contrary, the Cel@M2‐L‐MSN group exhibited significantly recovered collagen deposition, which was close to that of healthy rats. The above histological analyses showed that Cel@M2‐L‐MSN substantially inhibited synovial hyperplasia, attenuated inflammation, and promoted cartilage regeneration.

Additionally, micro‐computed tomography (Micro‐CT) was utilized for bone erosion assessments in different rat groups [38, 66]. The ankle joints of AIA rats exhibited tremendous bone damage and cortical discontinuity (Figure 6F), accompanied by significantly decreased bone mineral density (BMD), bone volume fraction (BV/TV), trabecular number (Tb.N), trabecular thickness (Tb.Th), and tissue mineral density (TMD), but dramatically increased trabecular spacing (Tb.Sp), structure model index (SMI), and total porosity compared with the control group (Figures 6G–I, S13). In contrast to severe bone degeneration in the model group, ameliorated bone integrity was observed within the Cel formulation groups. Importantly, Cel@M2‐L‐MSN effectively mitigated bone loss as evidenced by minimal bone microarchitecture changes and dramatically reversed BMD, BV/TV, Tb. N, Tb.Th, TMD, Tb.Sp, SMI, and total porosity levels to those of healthy rats.

### 2.8 Reshaping the RA Microenvironment

**FIGURE 7:**
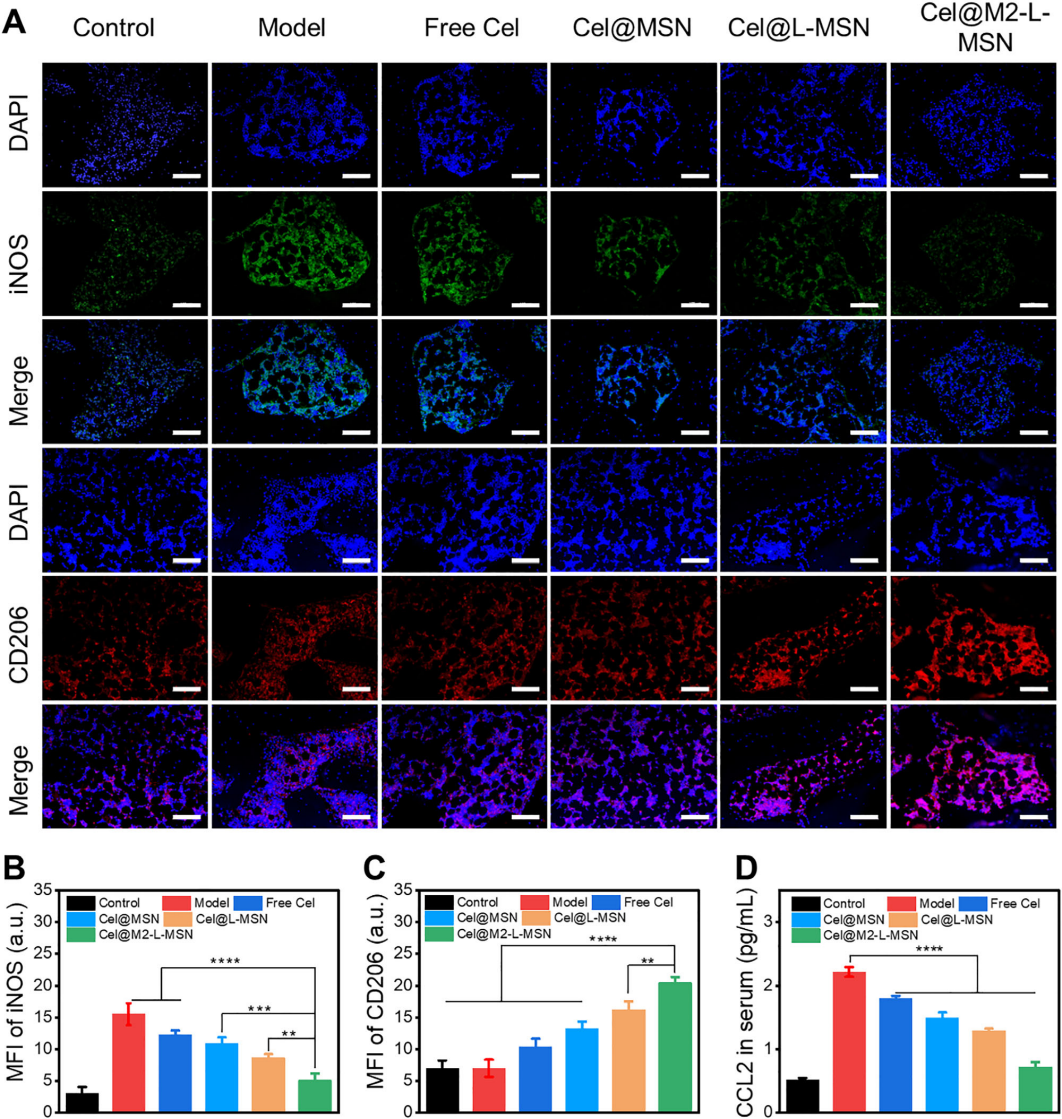
Macrophage repolarization and CCL2 blocking effects of Cel@M2‐L‐MSN in arthritic joints. (A) Immunofluorescence staining of macrophages with M1 (green for iNOS) and M2 (red for CD206) markers in the joints of various groups. Quantification of (B) iNOS, (C) CD206 levels in synovium tissues, and (D) CCL2 levels in serum after different treatments. Scale bar, 100 µm. Data were expressed as mean ± SD with *n* = 6. ^∗∗^ *p* < 0.01, ^∗∗∗^ *p* < 0.001, and ^∗∗∗∗^ *p* < 0.0001.

Immunostaining and immunohistochemical analysis, along with ELISA tests, were further performed so as to investigate the remodeling capabilities of Cel@M2‐L‐MSN on the complex microenvironment in arthritic joints. First, macrophage phenotypes were assessed through immunostaining with iNOS and CD206, respectively, as M1 and M2 macrophage markers (Figures 7A–C) [67]. Resultantly, the model group demonstrated intense fluorescence signals of iNOS (green) but negligible intensity of CD206 (red), suggesting a predominant phenotypic transition of macrophages towards the inflammatory M1 phenotype. In contrast, treatment with Cel formulations resulted in varying degrees of reversal of macrophage polarization. Remarkably, Cel@M2‐L‐MSN exhibited the most prominent increase in CD206 expression but downregulation of iNOS expression, thereby successfully repolarizing the macrophages to the M2 phenotype.

In RA, chemokines are highly produced to perpetuate local and systemic inflammation, which is intimately regulated by interactions between chemokine ligands and receptors [68]. Therefore, targeting chemokines can provide therapeutic benefits for RA remission. CCL2, alternatively known as monocyte chemotactic protein 1 (MCP‐1), not only promotes monocyte/macrophage infiltration but also enhances osteoclast formation and bone resorption. Consequently, the inhibition of the CCL2/CCR2 signaling pathway has been widely recognized as a therapeutic target to combat RA [69]. In line with previous reports, there was a significant increase in serum levels of CCL2 chemokines in AIA rats, which were 4.3‐fold that of the control group (Figure 7D). Cel formulations, namely, free Cel, Cel@MSN, Cel@L‐MSN, and Cel@M2‐L‐MSN, respectively, reduced CCL2 expression to 80.9%, 67.3%, 58.4%, and 32.4% that of the non‐treated AIA rats. Therefore, Cel@M2‐L‐MSN can serve as CCL2/CCR2 blocking agents for RA therapy.

**FIGURE 8:**
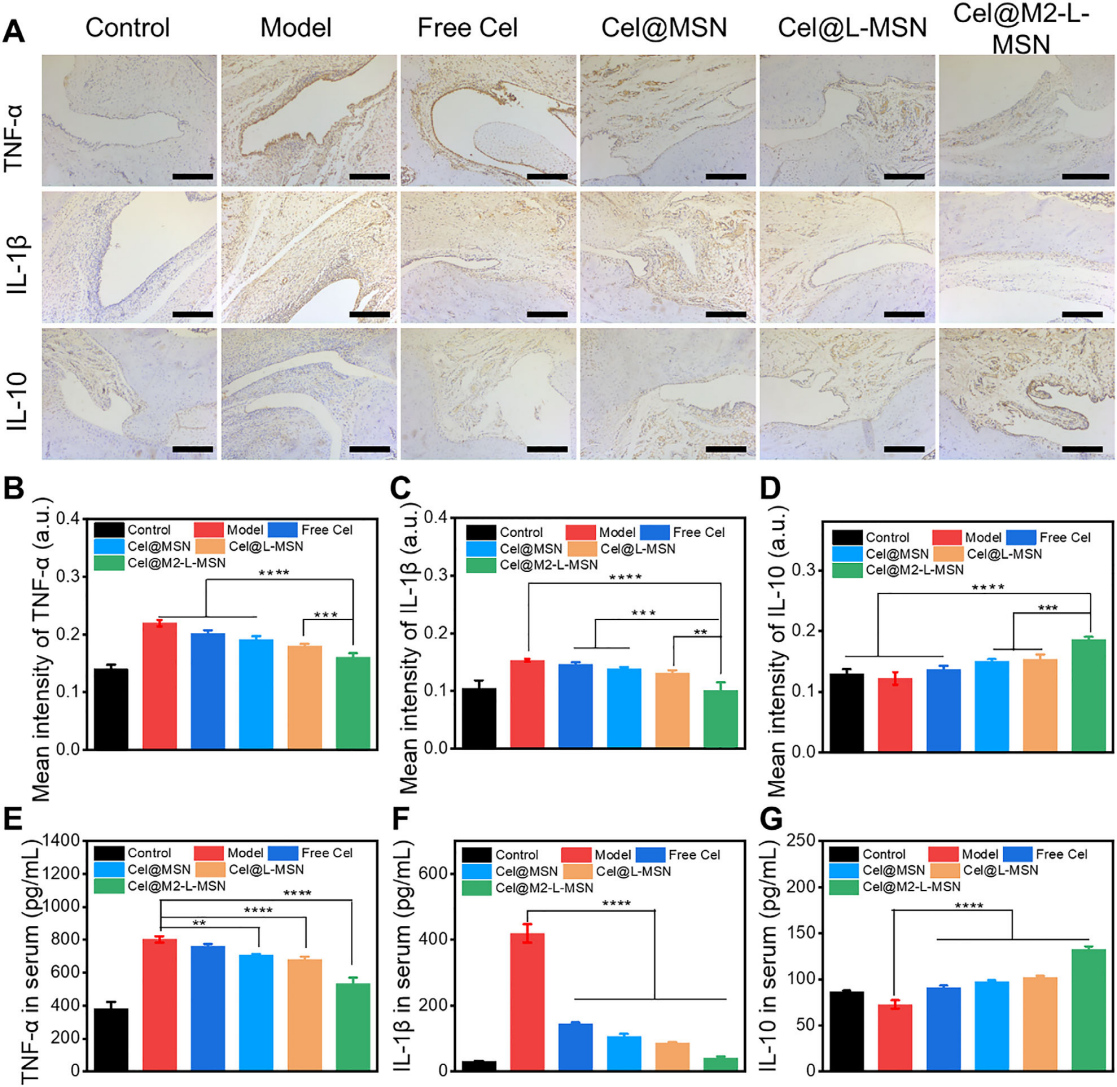
Inflammation downregulation effects of Cel@M2‐L‐MSN. (A) Immunohistochemical staining and corresponding quantification of (B) TNF‐α, (C) IL‐1β, and (D) IL‐10 levels in the synovial tissues after various treatments. (E) TNF‐α, (F) IL‐1β, and (G) IL‐10 expressions in the serum of rats from various groups. Scale bar, 100 µm. Data were presented as mean ± SD with *n* = 6. ^∗∗^ *p* < 0.01, ^∗∗∗^ *p* < 0.001, and ^∗∗∗∗^ *p* < 0.0001.

Meanwhile, immunohistochemical studies demonstrated that RA progression in the model group gave rise to high production of proinflammatory cytokines (TNF‐α and IL‐1β) but low anti‐inflammatory cytokine IL‐10 levels in the joints (Figure 8A–D). In comparison, Cel@M2‐L‐MSN outperformed the other Cel formulations and tremendously downregulated the expression of TNF‐α and IL‐1β while significantly upregulated IL‐10 expression, which mitigates immune responses and facilitates tissue repair. Furthermore, the expressions of TNF‐α, IL‐1β, and IL‐10 in serum were in accordance with the immunohistochemical analyses (Figure 8E–G). Hence, Cel@M2‐L‐MSN demonstrated potent immunoregulatory effects both locally and systemically, leading to the successful alleviation of RA symptoms.

### 2.9 Preliminary Biosafety In Vivo

To assess the biocompatibility of Cel@M2‐L‐MSN, the serological analysis, organ index of AIA rats, and pathological alterations in vital organs were monitored at the end of the pharmacodynamic evaluation. The model group showed elevated concentrations of ALT, AST, CRE, and BUN compared to healthy rats, especially for ALT and BUN, which suggested hepatic and renal dysfunction. On the contrary, the administration of Cel@M2‐L‐MSN normalized the levels of CRE, AST, ALT, and BUN, indicating recovered liver and kidney functions (Figures S14A–D). Additionally, there was a lack of significant differences in the indexes of major organs between the Cel@M2‐L‐MSN group and healthy rats (Figures S12, S15A–D). Furthermore, the H&E staining of major organs, namely the heart, liver, spleen, lung, and kidney, disclosed no evident pathological changes in the Cel@M2‐L‐MSN group compared with the control group (Figure S16). These results indicated that Cel@M2‐L‐MSN can serve as a safe nanomedicine for clinical RA management.

## 3 Conclusions

In summary, we established a biohybrid and ROS‐sensitive nanosystem (Cel@M2‐L‐MSN) for efficient RA treatment by fusing an anti‐arthritic agent, Cel‐loaded and lipid‐sealed MSN nanocore, and M2 macrophage membrane vesicles. The M2 macrophage membrane facilitated the targeted accumulation of Cel@M2‐L‐MSN in the RA joint for microenvironment remodeling, while the lipid potentiated ROS‐responsive drug release and avoided premature leakage in non‐targeted sites. Taking advantage of the combinatorial design and assembly, Cel@M2‐L‐MSN was anticipated to alleviate RA by simultaneously attenuating inflammation responses, scavenging ROS, inhibiting FLS migration and invasion, and promoting macrophage repolarization. Consequently, Cel@M2‐L‐MSN successfully alleviated joint destruction and organ dysfunctions in AIA rats, as evidenced by systematic pharmacodynamic investigations. Meanwhile, Cel@M2‐L‐MSN exhibited desirable biocompatibility both i*n vitro* and in vivo. Therefore, the as‐established Cel@M2‐L‐MSN confers a promising prospect for the clinical management of an array of inflammation‐dominated disorders, including RA.

## 4 Experimental Section

Detailed materials and methods are supplemented with supporting information.

## Conflicts of Interest

The authors declare no conflicts of interest.

## Supporting information




**Supporting File**: exp270213–sup‐0001‐SupMat.docx.

## Data Availability

The data that support the findings of this study are available from the corresponding author upon reasonable request.
