# Supplementary material for: M2 Macrophage‐Mimetic and ROS‐Sensitive Hybrid Nanoplatform for Targeted Alleviation of Rheumatoid Arthritis
Source: Exploration (Beijing). 2026 Aug 19;6(4):20230030. doi: 10.1002/EXP.20230030 (PMC13490676; doi:10.1002/EXP.20230030)
Supplement: Supplementary file 1 [file EXP2-6-20230030-s001.docx]

Supporting Information

**M2 Macrophage-Mimetic and ROS-Sensitive Hybrid Nanoplatform for Targeted Alleviation of Rheumatoid Arthritis**

*Yi Xiao ^a^, Shiyu Meng ^a^, Zhuang Tang ^a^, Yonghang Liu ^a^, Xican Zhang ^a^, Kefan Wu ^a^, Yuanhao Liu ^a^, Ao Li ^a^, Jingyao Feng ^a^, Kaixiang Zhou ^b^, Hui Guo ^a, c *^, Xiaolin Wang* *^a *^*

*^a^* School of Pharmacy and State Key Laboratory of Quality Research in Chinese Medicine, Macau University of Science and Technology, Macao 999078, China

*^b^* Center for Advanced Materials Research, Advanced Institute of Natural Sciences, Beijing Normal University at Zhuhai, Zhuhai 519087, China.

*^c^* School of Chemical Engineering and Technology, Sun Yat-sen University, Zhuhai 519082, China

*Corresponding author Email:

guoh37@mail.sysu.edu.cn (H. G.)

[xilwang@must.edu.mo](mailto:xilwang@must.edu.mo) (X. W.)

**EXPERIMENTAL SECTION**

**Materials**

Tetraethyl orthosilicate (TEOS), cetyltrimethylammonium bromide (CTAB), and (3-aminopropyl) triethoxysilane (APTES) were purchased from Macklin Co., Ltd (Shanghai, China). DOPC (Catalog No. LP-R4-070) and DSPE-PEG-2000 (Catalog No. LP-R4-039) were purchased from Corden Pharma Switzerland (Liestal, Switzerland). Celastrol (Lot) was purchased from Dalian Bergolin Biotechnology Co., Ltd (Dalian, China). Anti-LFA-1 antibody (Catalog No.ET1610-1), Anti-CCR2 antibody (Catalog No. ET1611-65), and anti-IL-1R antibody (Catalog No. EM1708-33) were purchased from Hangzhou HuaAn Biotechnology Co., Ltd (Hangzhou, China). Anti-TNF Receptor antibody (Catalog No. ab259817) was purchased from Abcam (MA, USA). Chloroquine (Catalog No. 1118000) and cholesterol (Catalog No. C8667-5G) were purchased from Sigma (MO, USA). Filipin (Catalog No. HY-N6716) and Cy5-SE (HY-D0819) were purchased from MedChemExpress (New Jersey, USA). Anti-ICAM-1 antibody (Catalog No. sc-8439) and Anti-GAPDH antibody (Catalog No. sc-365062) were purchased from Santa Cruz Biotechnology (CA, USA). Recombinant murine IL-4 (Catalog No. 214-14), recombinant murine IL-13 (Catalog No. 210-13), recombinant murine TNF-α (Catalog No. 315-01A), recombinant murine IL-1β (Catalog No. 211-11B), and recombinant murine CCL2 (Catalog No. 250-10) were purchased from PeproTech (Rocky Hill, NJ, USA). Coomassie Blue Staining Solution (Catalog No. cw0023s) was purchased from Cwbio IT Group (Beijing, China). Fluorescein isothiocyanate isomer (FITC) was purchased from Sigma-Aldrich Inc. (St. Louis, MO, USA). DiD (1,1′-dioctadecyl-3,3,3′,3′-tetramethylindodicarbocyanine perchlorate) (Catalog No. D307), lipopolysaccharide (LPS), DCFHDA (2',7'-dichlorodihydrofluorescein diacetate) (Catalog No. D399), and DAPI (4,6-diamidino-2phenylindole) (Catalog No. 62488) were purchased from Thermo Fisher Scientific (MA, USA). TNF-α rabbit polyclonal antibody (GB11188), IL-10 rabbit polyclonal antibody (GB11117), and IL-1β rabbit polyclonal antibody (GB11113) were purchased from ServiceBio Technology Company (Wuhan, China). The dialysis bag (MWCO 8000-14000) (Catalog No. 92420602) was purchased from Sinopharm Chemical Reagent (Shanghai, China). Alamar Blue (MX3006) was purchased from Maokangbio (Shanghai, China). Rat TNF-α ELISA Kit (Catalog No. EK382/3), Rat IL-10 ELISA Kit (Catalog No. EK310/2), Rat IL-1β ELISA Kit (Catalog No. EK301B/3), and Rat CCL2 ELISA Kit (Catalog No. EK387) were purchased from Multi Sciences (Hangzhou, China). Mouse TNF-α ELISA Kit (Catalog No. EK282/4), Mouse IL-1β ELISA Kit (Catalog No. EK201B/3), and Mouse CCL2 ELISA Kit (Catalog No. EK287/2) were purchased from Multi Sciences (Hangzhou, China). Glutamic-pyruvic transaminase (ALT) test kit (Catalog No. C009-2-1), Aspartate aminotransferase (AST) test kit (Catalog No. C010-2-1), Blood urea nitrogen (BUN) test kit (Catalog No. C013-2-1), and Creatinine (CRE) test kit (Catalog No. C011-2-1) were purchased from Nanjing Jiancheng Bioengineering Institute (Nanjing, China). Dulbecco's modified Eagle medium (DMEM), fetal bovine serum (FBS), penicillin-streptomycin stock solutions, and trypsin-EDTA (0.25%) were obtained from Invitrogen (CA, USA).

**Methods**

**Cell Culture**

MH7A cells and RAW 264.7 cells were purchased from the Chinese Academy of Sciences Cell Bank (Shanghai, China). MH7A cells and RAW 264.7 cells were cultured in Dulbecco's modified Eagle medium (DMEM) with 10% FBS and 1% Penicillin-Streptomycin Solution. Both cell lines were cultured at 37 °C in a cell incubator with 5% CO_2_.

**M2 Macrophage Membrane Derivation**

RAW264.7 cells were induced with 10 ng/mL IL-4 and 10 ng/mL IL-13 for 24 h to obtain M2 macrophages.^[1]^ RAW264.7 cells without treatment were considered naïve macrophages (M0) as the control group. The upregulation of Arg-1 expression in M2 macrophages was confirmed by western blotting and ImageJ analysis.

Subsequently, the M2 macrophage membrane was deprived following previous reports.^[2]^ More specifically, M2 macrophages were suspended at a density of 2.0 × 10^7^ cells/mL in TM buffer (pH = 7.4, 10 mM Tris + 1 mM MgCl_2_) and subsequently homogenized with a Dounce homogenizer 100 times. The resulting cell homogenate was mixed with sucrose solution to reach a final concentration of 0.25 M sucrose before centrifuging at 2000 ×g and 4 ℃ for 10 mins. Afterward, the supernatant was collected and subjected to centrifugation at 3000 ×g for another 30 min. Finally, the precipitate was collected and washed with a mixture of TM buffer and 0.25 M sucrose twice to obtain purified M2 macrophage membranes. The bicinchoninic acid assay (BCA) protein assay (Beyotime, China) was employed to analyze the total protein content in the purified M2 macrophage membrane. Approximately 100 million RAW264.7 cells were able to yield 0.4 mg of membrane material (total protein weight). The as-obtained M2 macrophage membranes were stored at −80 °C before use.

**Synthesis of MSN, L-MSN, and M2-L-MSN and fluorescence labeling**

Bare MSNs were synthesized according to the literature with minor modifications ^[3]^. In brief, cetyltrimethylammonium bromide (CTAB, 0.40 g) was dissolved in 200 mL of deionized water, followed by the addition of 1.44 mL of NaOH solution (2.0 M) and 0.4 mL of ethanol, and heating to 80˚C under stirring. Afterward, 2 mL tetraethoxysilane (TEOS) was introduced dropwise to the solution, and the mixture was allowed to stir for 2 h, giving rise to a white precipitate. The solid product was filtered, washed with deionized water and methanol, and dried in an oven overnight. Removal of the CTAB surfactant template was performed by calcination at 550 ˚C for 5 h.

To obtain lipid layer-capped MSNs (L-MSN), 10 mg of solids consisting of DOPC, cholesterol (Chol), and DSPE-PEG2000 at a molar ratio of 58: 38: 5 were deposited in a 100 mL flask and dissolved in a mixture of chloroform and methanol (3:1, v/v). After the removal of the organic solvent by rotary evaporation, a thin lipid film was formed on the inner wall of the flask, which was hydrated with 5 mL of MSN suspension (2 mg/mL) and sonicated to allow for homogeneous lipid wrapping over the nanosurface of MSNs. Finally, the as-obtained L-MSN was fused with the M2 macrophage membrane to assemble into M2-L-MSN.^[4]^ To this end, 2 mg M2 macrophage membrane was dispersed in 2 mL PBS, mixed with 2 mg L-MSN, and sonicated in an ice bath for 2 min. Purified M2-L-MSN was obtained after removing the uncoated M2 membrane by centrifugation at 12000 rpm for 10 min.

For fluorescence labeling, 50 mg MSN was first dispersed in methanol (100 mL), and APTES (0.4 mL) was dropwise added for stirring overnight. Next, the suspension was centrifuged and washed with methanol to obtain amino-conjugated nanoparticles (MSN-NH_2_). Subsequently, FITC (0.3 mg) was mixed with MSN-NH_2_ (30 mg) in 3 mL ethanol and stirred overnight to generate FITC-labeled nanoparticles (MSN-FITC). Cyanine 5.5-doped MSNs (MSN-Cy5) were prepared in the same way except that Cy5 was used in place of FITC. Moreover, MSN labeled with FITC or Cy5 were further functionalized with a lipid layer and M2 macrophage membrane vesicles as described above to generate fluorescent L-MSN and M2-L-MSN.

**Characterization of Nanoparticles**

The morphology of nanoparticles was observed by Transmission electron microscopy (TEM) (FEI Talos F200X, USA). The hydrodynamic particle sizes and zeta potentials of MSN, Cel@MSN, Cel@L-MSN, and Cel@M2-L-MSN were determined by Zetasizer Nano ZS (Malvern, UK). Nitrogen adsorption-desorption measurements were performed on an ASAP 2020 instrument (Micromeritics, USA) after degasification, and the surface area, pore volume, and pore size were obtained by the Brunauer–Emmett–Teller (BET) and Barrett–Joyner–Halenda (BJH) analyses, respectively. XRD patterns of the samples were collected using an X-ray diffractometer equipped with a CuKα target (D8 Advance, Bruker, Germany).

**Identification of M2 Macrophage Membrane Proteins**

Western blotting was employed to detect proteins expressed in both differentiated and undifferentiated RAW 264.7 cells, as well as to verify the characteristic M2 macrophage membrane proteins. To obtain M2 phenotype macrophages, RAW 264.7 cells were differentiated with IL-4 (10 ng mL^-1^) and IL-13 (10 ng mL^-1^) for 24 h, followed by rinsing with ice-cold PBS 3 times at 800 rpm for 10 min. Subsequently, activated RAW 264.7 cells were lysed using RIPA lysis buffer for 30 min, centrifuged (12,000 g, 5 min, 4 °C), and the supernatant was collected. The total protein in the supernatant was quantified using a BCA protein assay. The total protein in the supernatant was mixed with 5× loading buffer, and the obtained protein samples were heated at 100 °C for 5 min. The protein samples of Cel@M2-L-MSN, M2, and MSN were prepared similarly. Based on the instructions from the manufacturers, samples (20 μg/ well) were loaded into the SDS-PAGE gel and separated by a MiniPROTEAN Tetra System (BIO-RAD, CA, USA). Afterward, the samples were stained with Coomassie Blue, destained with water overnight, and eventually imaged.

The Bio-Rad TGX Fast-Cast acrylamide kit was prepared for the SDS-PAGE gels. The upper gel ran at 80 V, and the lower gel ran at 120 V to separate proteins. When the electrophoresis was finished, the proteins isolated were removed from the SDS-PAGE gel on a glass plate and transferred to the transfer buffer. The SDS-PAGE gel was held as a sandwich in the following order: whiteboard, sponge, filter paper, Gel, PVDF membrane, filter paper, sponge, and blackboard. Insert the gel exactly into the transfer tank. The transfer tank was buried in the ice wrapped in ice. The protein was transferred to the activated PVDF membrane (parameters were set as 300 mA, 30 min to 1 h). After the protein was transferred, the PVDF membrane was blocked with 5% skim milk at room temperature for 1 h. PVDF membranes were incubated with corresponding primary antibodies Arg-1, Tubulin, LFA-1, IL-1R, TNF-αR, and CCR2 overnight at 4 °C. After the PVDF membranes were washed 3 times, the membranes were then incubated with the corresponding secondary antibody (1:5000) for 60 min at room temperature. Then, the PVDF was washed 3 times. Then, solution A and solution B in the ECL kit were mixed at a ratio of 1:1 to obtain the detection solution. The detection solution was added to the PVDF membrane. The proteins were visualized with a chemiluminescence imaging system. The grey values of the obtained images were analyzed with ImageJ.

**Colocalization Assay**

To validate successful macrophage membrane camouflaging, M2-DID-L-MSN-FITC was prepared by fusing M2 macrophage membrane stained with 1,1″-octacosyl-3,3′, 3′tetramethyl indole dicarbocyanine perchlorate (DiD, excitation/ emission = 644/665 nm) and L-MSN labeled with FITC. Afterward, RAW264.7 cells were cultured in a 24-well plate at a density of 1 × 10^4^ and activated with 100 ng/mL LPS overnight. Next, M2-DID-L-MSN-FITC (30 μg/mL) was added and incubated at 37 °C for 4 h. Subsequently, RAW264.7 cells were fixed with 4% PFA for 10 min, DAPI-stained for 10 min, and images were obtained by confocal microscopy (Leica TCS SP8, Germany).

**Drug Loading**

Cel was successfully loaded into MSNs by solvent impregnation. Briefly, 30 mg of MSN and 15 mg of Cel were mixed in 2 mL of dichloromethane and stirred for 24 h. Then, the nanoparticles were collected and washed by centrifugation and dried at room temperature to obtain Cel@MSN, which was further functionalized with a lipid layer or together with M2 macrophage membranes to obtain Cel@L-MSN or Cel@M2-L-MSN. To evaluate the drug-loading capacity of Cel@M2-L-MSN, the supernatant after each centrifugation was collected to detect free Cel by UV-2700 (UV-vis spectrophotometer, Shimadzu) at a wavelength of 425 nm. Drug loading efficiency (LE%) and encapsulation efficiency (EE) were calculated by the following equations.

$$LE \left( \% \right)= \frac{Weight of total drug-Weight of free drug}{Weight of nanoparticles}\times100\%$$

$$EE \left( \% \right)=\frac{Weight of total drug-Weight of free drug}{Weight of total drug}\times100\%$$

**Drug Release Kinetics**

The drug release kinetics of free Cel, Cel@MSN, Cel@L-MSN, and Cel@M2-L-MSN were evaluated in phosphate-buffered saline (PBS, pH = 7.4) containing 0.2% SDS using Spectra/Por 3500 Da-MWCO membrane tubing, which was kept in water bath at 37 °C with a shaking speed of 100 rpm. 2 mL of the release medium were collected at predetermined time intervals and replenished with an equal volume of fresh buffer.

Simvastatin (SIM) instead of Cel was loaded into L-MSN to study the ROS-triggered drug release behaviors of the M2 macrophage-mimetic nanoparticles. SIM@M2-L-MSN was loaded into dialysis bags with a molecular weight cut-off of 3500 Da (Solarbio, China) and dialyzed in 40 mL of different media at 37 °C with constant shaking at 100 rpm. At predetermined time points, an aliquot (2 mL) of the release medium was withdrawn while the same volume of fresh medium was replenished. The concentration of Cel in the release medium was determined by UV spectrophotometry, and the cumulative release rate was calculated by the following equation:

$$Cumulative release \left( \% \right)=\frac{M_{t}}{M_{0}}\times100\%$$

where M_t_ stands for the quantity of drug released at time t, and M_0_ is the total amount of drug in the free form or loaded in the nanoparticles.

**Cellular Uptake**

MH7A cells were cultured in a 24-well plate at 37 °C and activated by 100 ng/mL LPS overnight. Subsequently, 30 μg/mL MSN-FITC, L-MSN-FITC, M2-MSN-FITC, and M2-L-MSN-FITC were added to the wells and incubated for another 4 h. After removal of the supernatant, the cells were washed and fixed with 4% PFA for 10 min and stained with DAPI for 10 min. Then, the cells were observed by a confocal microscope. Moreover, cellular uptake was conducted in macrophages and LPS-treated macrophages.^[5]^ After seeding the cells and incubating at 37 °C for 24 h, the medium was replaced with fresh medium containing MSN-FITC and M2-L-MSN-FITC, and the cells were incubated for an additional 1–4 h, respectively. Subsequently, the cells were rinsed and fixed with 4% PFA for 10 min and stained with DAPI for 10 min. The cells were observed by CLSM. To verify M2-mediated cellular uptake, ICAM-1 expression on MH7A cells and RAW 264.7 cells with or without LPS treatment was evaluated by Western blotting.

Next, the mechanism underlying the cellular uptake was explored. MH7A cells and RAW264.7 cells activated with 100 ng/mL LPS overnight were pretreated with chlorpromazine (CPZ, 10 μg/mL), filipin (1 μg/mL), sodium azide (NaN_3_, 1 mg/mL), and cytochalasin D (Cyto-D, 1.5 μg/mL) for 1 h, followed by incubation with M2-L-MSN-FITC for another 4 h.^[6]^ The cell nuclei were stained with DAPI, and the cells were observed by CLSM.

**Cytokines and Chemokines Binding Capability**

Cel@M2-L-MSN was diluted to a final concentration of 0~2048 μg/mL. After incubation with TNF-α, IL-1β, and CCL2 at 37 °C for 1 h, the nanosystem was removed by centrifugation at 4 °C at 12 000 rpm for 30 min.^[7]^ The supernatant containing TNF-α, IL-1β, and CCL2 was quantified by ELISA tests.

**ROS Scavenging *In Vitro***

RAW 264.7 cells were cultured in 6-well plates at a density of 1 × 10^6^ cells per well and stimulated with LPS as mentioned above. After the culture medium was removed, the cells were stained with 10 μM DCFH-DA for 30 min.^[8]^ The well without LPS was used as the negative control, while cells treated with LPS alone were used as the positive control.

**Migration and Invasion Assay**

MH7A cells were seeded into a 6-well plate. 5 of these wells were activated for 12 h with LPS (1 μg/mL) when the cells reached 80–90% confluence. Next, a straight line was scraped with the pipette tip in a 6-well plate, rinsed with PBS, and cultured in a serum-free medium. Subsequently, free Cel, Cel@MSN, Cel@L-MSN, and Cel@M2-L-MSN (equivalent to 25 ng/mL Cel) were added and incubated for 24 h. The microscope (CKX53, Olympus, Japan) was used to observe and photograph the scratch area, and the relative migration area was calculated as follows:

$$Relative migration area \left( \% \right)=\frac{A_{0 h}-A_{24 h}}{A_{0 h}}\times100\%$$

where A_0 h_ and A_24 h_ stand for the scratch area at 0 h and 24 h, respectively.

For the invasion assay, MH7A cells were activated by 1 μg/mL LPS for 12 h before the transwell inserts (pore size of 8 μm) were coated with Matrigel at 37 °C for 1 h. Subsequently, activated MH7A cells were harvested, inoculated into the transwell inserts with serum-free medium, and co-incubated with free Cel, Cel@MSN, Cel@L-MSN, and Cel@M2-L-MSN (equivalent to 25 ng/mL Cel) for 24 h, while the bottom chambers were filled with the complete medium containing 10% FBS. Afterward, the cells on the upper side of the insert membrane were removed with a cotton swab, while those on the opposite side were fixed with 4% PFA and stained with 0.1% crystal violet. The number of invasive cells was quantified in three random fields under a light microscope.

**Macrophages Polarization *In Vitro***

RAW264.7 cells were seeded into 6-well plates at a density of 1×10^6^ cells/mL and divided into the control, LPS, free Cel, Cel@MSN, Cel@L-MSN, and Cel@M2-L-MSN groups. LPS (100 ng/mL) was added for 30 minutes of incubation, except for the control group. Then, the cells were treated with fresh complete culture medium containing Cel or Cel@MSN or Cel@L-MSN or Cel@M2-L-MSN at an equivalent dose of 100 ng/mL Cel for 24 h.^[9]^ Afterward, RAW264.7 cells were incubated with CD16/32 for 10 min to block Fc receptors, followed by staining with PerCP/Cyanine5.5 anti-mouse CD86 antibody and PE anti-mouse CD206 antibody for 30 min and detection using flow cytometry.

**TNF-α and IL-1β Expression *In Vitro***

The cell supernatants of the above groups were aspirated, and then the levels of cytokines (TNF-α and IL-1β) in the cell supernatants of each group were detected with an ELISA kit following the manufacturer’s instructions.

**Cytotoxicity and Hemolysis Assay**

Cell viability was determined by the Alamar Blue™ test.^[10]^ MH7A cells and RAW264.7 were seeded in 96-well plates (1×10^4^ cells per well) and incubated for 24 h. Then, free Cel, Cel@M2-MSN, and Cel@M2-L-MSN at different concentrations (50, 100, 200, 400, 600, and 800 ng/mL) were seeded into the 96-well plate, respectively. After 24 h co-incubation, the cell culture medium was replaced with that containing 10% Alamar Blue and incubated for another 4 h. The optical density (OD) was measured at 570 nm and 600 nm by the microplate reader (SpectraMax ID5, Molecular, USA). The cell viability of each group was calculated according to the formula in the instruction manual and expressed as a percentage of the control group.

Fresh red blood cells (RBCs) were collected from healthy SD rat whole blood by centrifuging and diluted to a 2% (v/v) erythrocyte suspension with PBS. Cel@M2-L-MSN, Cel@MSN, and MSN were diluted at different concentrations of 20, 50, 100, 200, 400, and 600 μg/mL and co-incubated with the RBC suspension. Distilled Milli-Q water was used as the positive control, and PBS was used as the negative control. The mixture was incubated for 3 h at 37 °C and centrifuged. The absorbance of the supernatant was determined at 540 nm by the microplate reader (n = 3). The hemolysis rate of the RBCs was calculated as follows:

$$Hemolysis \left( \% \right)=\frac{Asample-Anegative}{Apositive-Anegative}\times100\%$$

**AIA model establishment**

SPF-grade male Sprague-Dawley (SD) rats (180 ± 20 g) were purchased from SPF Biotechnology Co., Ltd, Beijing, China. All the animal study protocols were approved by the Ethics Committee of the Animal Center of Macau University of Science and Technology (MUST-MOST-2021072001). The adjuvant-induced arthritis (AIA) rat models were established as previously reported.^[11]^ In brief, 100 μL of complete Freund’s adjuvant emulsion containing 150 μg/mL heat-inactivated Mycobacterium tuberculosis (Mtb) was injected subcutaneously at the base of the tail of SD rats.

**Pharmacokinetic Study**

The AIA rats were intravenously injected with free Cy5 and Cy5-labeled MSN, L-MSN, and M2-L-MSN (at an equivalent Cy5 dose of 5 μg/kg). The blood of rats was collected at 0.5, 1, 2, 4, 6, 12, and 24 h after injection, transferred into 96-well plates, and photographed by an in vivo imaging system (IVIS, Caliper, USA).

**Biodistribution *In Vivo***

The AIA rat model was established in SD rats as mentioned in the previous section. 12 days post-modeling, free Cy5 and Cy5-labeled MSN, L-MSN, and M2-L-MSN were administered to AIA rats by intravenous injection (at an equivalent Cy5 dose of 5 μg/kg). Fluorescence images in vivo of the hind paws were captured at 2, 6, 12, and 24 h using an in vivo imaging system (IVIS, Caliper, USA). Meanwhile, the major organs, including the heart, liver, spleen, lung, kidney, and joint tissues of the rats, were collected for imaging and biodistribution analysis.

**Anti-RA Treatments *In Vivo***

The AIA rats were randomly assigned into five groups (6 rats for each group) 12 days after model induction. Subsequently, the AIA model, free Cel, Cel@MSN, Cel@L-MSN, and Cel@M2-L-MSN (equivalent Cel dose at 1 mg/kg) were intravenously administered to the AIA rats every two days from day 12 to day 28. The body weight, arthritic score, and hind paw volume of rats in different groups were monitored every 3 days for 30 days. Besides, the arthritic score was evaluated every 3 days. The scoring criteria were as follows: 0 = normal; 1 = erythema and mild swelling limited to the ankle joint; 2 = erythema and mild swelling extending from the ankle to the tarsals; 3 = erythema and moderate swelling extending from the ankle to metatarsal joints; and 4 = erythema and severe swelling encompassing the ankle, feet, and digits, or ankylosis of the limb.^[12]^ All rats on the 30th day after the arthritic induction were euthanized, and hind paws were photographed and collected for further investigations.

**Micro-CT Analysis**

Dissected ankle joints were analyzed by an *in vivo* micro-CT scanner (SkyScan1176, Bruker, Belgium) with the following parameters: current 294 μA, voltage 85 kV, exposure time 35 ms, resolution 35 μm, and an aluminum filter 1 mm.^[13]^ The visual 3D images were reconstructed using CT-Vol software (Bruker-micro-CT, Belgium), while the photos were reconstructed by NRecon software. The morphometric parameters of trabecular bone were determined using the CTAn program (Skyscan, Bruker, Belgium).

**Histopathology of inflamed joints**

On day 30, the hind paws of each group were collected and treated as previously reported. ^[14]^ More specifically, the ankle joints were dissected, fixed in 4% PFA, and decalcified in formalin-EDTA solution for 30 days at 25 °C. Afterward, the as-obtained joints were included in paraffin blocks, cut into thin sections with a thickness of 5 µm, and stained with hematoxylin and eosin (H&E), saffron O-fast green (SOFG), and Masson’s trichrome (Masson), respectively. The stained sections were observed and photographed under a microscope.

**Macrophages Polarization *In Vivo***

To analyze the macrophage phenotypes, the synovial tissue sections (thickness = 5 μm) of the rats were incubated with anti-CD206 and anti-iNOS antibodies at 4 °C overnight.^[15]^ Next, the slices were incubated with a secondary antibody combined with Alexa Fluor 488 and Cy3 for 60 min, respectively. Subsequently, the slices were stained with DAPI for 5 min before observation with CLSM.

**Immunohistochemical Analysis**

The ankle joints retrieved from the rats were also subjected to immunohistochemical analysis after routine fixation, decalcification, paraffin embedding, and cutting as described above. After dewaxing, tissue sections were sequentially incubated with specific antibodies against rat TNF-α, IL-1β, and IL-10, followed by incubation with appropriate secondary antibodies. Finally, the expressions of TNF-α, IL-1β, and IL-10 in the joint tissue were captured by a light microscope and quantitatively analyzed by ImageJ.

**Serum Inflammatory Cytokines and Chemokines Detections**

The blood of the healthy and AIA rats was collected on day 30, and the serum levels of TNF-α, IL-1β, IL-10, and CCL2 were measured by the ELISA kits following the instructions of the manufacturers.

**Safety Evaluations *In Vivo***

To evaluate the safety of the biomimetic nanoparticle, major organs including the heart, liver, spleen, lung, and kidney were collected from the rats of different groups, embedded in paraffin, cut into 5 μm sections, stained with H&E, and analyzed under a light microscope. Besides, the serum levels of alanine transaminase (ALT), aspartate aminotransferase (AST), creatinine (CRE), and blood urea nitrogen (BUN) were detected according to the kit instructions to explore potential liver and kidney injury. In addition, the weight of all the major organs was measured to calculate the corresponding organ index according to the following formula.

$$Organ index \left( \frac{mg}{g} \right)=\frac{Weight of organ (mg)}{Body Weight (g)}$$

**Statistical Analysis**

All the data are expressed as mean ± SD. Student’s t-test (to compare the variances between two groups) or One-way analysis of variance (ANOVA) followed by Tukey’s multiple comparison post-hoc test (to compare the variances among three or more groups) was used for statistical analysis. P < 0.05 was deemed statistically significant.

**References**

[1]	K. Y. W. Teo, C. Sevencan, Y. A. Cheow, S. Zhang, D. T. Leong, W. S. Toh, Small Science 2022, 2, 2100116.

[2]	Y. Yue, F. Li, Y. Li, Y. Wang, X. Guo, Z. Cheng, N. Li, X. Ma, G. Nie, X. Zhao, ACS Nano 2021, 15, 15166.

[3]	S. Mu, Y. Liu, T. Wang, J. Zhang, D. Jiang, X. Yu, N. Zhang, Acta Biomater. 2017, 63, 150.

[4]	S. Wu, Y. Huang, J. Yan, Y. Li, J. Wang, Y. Y. Yang, P. Yuan, X. Ding, Adv Funct Mater. 2021, 31, 2103442.

[5]	C. Gao, Q. Huang, C. Liu, C. H. T. Kwong, L. Yue, J.-B. Wan, S. M. Y. Lee, R. Wang, Nat Commun. 2020, 11, 2622.

[6]	Z. Tang, S. Meng, X. Yang, Y. Xiao, W. Wang, Y. Liu, K. Wu, X. Zhang, H. Guo, Y. Z. Zhu, X. Wang, Small 2023, 2307379.

[7]	Z. Tang, S. Meng, X. Yang, Y. Xiao, W. Wang, Y. Liu, K. Wu, X. Zhang, H. Guo, Y. Z. Zhu, X. Wang, Small 2023, n/a, 2307379.

[8]	Y. Yang, L. Guo, Z. Wang, P. Liu, X. Liu, J. Ding, W. Zhou, Biomaterials 2021, 264, 120390.

[9]	N. Yang, M. Li, L. Wu, Y. Song, S. Yu, Y. Wan, W. Cheng, B. Yang, X. Mou, H. Yu, J. Zheng, X. Li, X. Yu, J Nanobiotechnol. 2023, 21, 13.

[10]	X. Wang, O. Ronsin, B. Gravez, N. Farman, T. Baumberger, F. Jaisser, T. Coradin, C. Hélary, Adv. Sci. 2021, 8, 2004213.

[11]	X. Chen, H. Zhang, W. Zeng, N. Wang, H. H. Lo, C. K. Ip, L. J. Yang, W. L. W. Hsiao, W. M. Sin, C. Xia, B. Y. K. Law, V. K. W. Wong, J Adv Res. 2022, 38, 107.

[12]	X. X. Fan, M. Z. Xu, E. L. H. Leung, C. Jun, Z. Yuan, L. Liu, Nano-Micro Lett. 2020, 12, 76.

[13]	S. Meng, Z. Song, Z. Tang, X. Yang, Y. Xiao, H. Guo, K. Zhou, M. Du, Y. Z. Zhu, X. Wang, Biomater Sci-Uk. 2023, 11, 7099.

[14]	H. Yu, J. Fan, N. Shehla, Y. Qiu, Y. Lin, Z. Wang, L. Cao, B. Li, M. Daniyal, Y. Qin, C. Peng, X. Cai, B. Liu, W. Wang, ACS Nano 2022, 16, 502.

[15]	B. Lu, C. Li, L. Jing, F. Zhuang, H. Xiang, Y. Chen, B. Huang, J Control Release 2023, 362, 631.

**List of Supplementary Figures:**

**Figure S1.** Nitrogen adsorption-desorption isotherms and pore size distribution of different nanoparticles.

**Figure S2.** Fourier transform infrared (FT-IR) spectra of Cel, MSN, and Cel@MSN.

**Figure S3.** XRD pattern of Cel, MSN, and Cel@MSN.

**Figure S4.** UV-Vis spectra of Cel, MSN, L-MSN, and M2-L-MSN in different media.

**Figure S5.** Western blot analysis of ICAM-1 expression in MH7A cells.

**Figure S6.** Western blot analysis of ICAM-1 expression in RAW264.7 cells.

**Figure S7.** Proinflammatory cytokines TNF-α and IL-1β expression levels in RAW264.7 cells.

**Figure S8.** Cytotoxicity of MSN, L-MSN, or M2-L-MSN at different concentrations in RAW264.7 cells.

**Figure S9.** Cytotoxicity of Cel and Cel@M2-L-MSN at different concentrations in MH7A cells.

**Figure S10.** Cytotoxicity of Cel and Cel@M2-L-MSN in RAW264.7 cells at different concentrations.

**Figure S11.** The hemocompatibility of MSN, Cel@MSN, M2-L-MSN, and Cel@M2-L-MSN at different concentrations.

**Figure S12.** Photographs of spleens from different groups and the corresponding spleen index.

**Figure S13.** Trabecular bone spacing (Tb.Sp), trabecular thickness (Tb.Th), cortical mineral density (TMD), structure model index (SMI), and total porosity of rats from different groups.

**Figure S14.** The levels of blood ALT, AST, BUN, and CRE from different groups.

**Figure S15.** The heart, liver, lung, and kidney index from different groups.

**Figure S16.** Representative H&E staining images of heart, liver, lung, and kidney tissue sections from different treatment groups.

**Figure S1.** (A) N_2_ adsorption-desorption isotherms and (B) pore size distribution of different nanoparticles.

**Figure S2.** Fourier transform infrared (FT-IR) spectra of Cel, MSN, and Cel@MSN.

**Figure S3.** XRD patterns of Cel, MSN, and Cel@MSN.

**Figure S4.** UV-Vis spectra of Cel, MSN, L-MSN, and M2-L-MSN in dichloromethane (CH_2_Cl_2_) or PBS (pH = 7.4).

**Figure S5.** (A) Expression of ICAM-1 in MH7A cells with (+) or without (-) treatment with LPS. Determined by Western blotting. (B) The corresponding quantitative analysis by ImageJ. Data were presented as mean ± SD for n = 3. ^∗∗∗^*p* < 0.001.

**Figure S6.** (A) Expression of ICAM-1 in RAW264.7 cells with (+) or without (-) treatment with LPS. Determined by Western blotting. (B) The corresponding quantitative analysis by ImageJ. Data were presented as mean ± SD for n = 3. ^∗∗∗^*p* < 0.001.

 **Figure S7.** Proinflammatory cytokines (A) TNF-α and (B) IL-1β expression levels in RAW 264.7 cells. Data were presented as mean ± SD for n = 3. ^∗∗^*p* < 0.01, ^∗∗∗^*p* < 0.001, ^∗∗∗∗^*p* < 0.0001.

**Figure S8.** Cytotoxicity of MSN, L-MSN, or M2-L-MSN in RAW264.7 cells at different nanoparticle concentrations. Data were presented as mean ± SD for n = 3.

**Figure S9.** Cytotoxicity of Cel, Cel@M2-MSN, or Cel@M2-L-MSN at various drug concentrations (50-800 ng/mL) in MH7A cells. Data were presented as mean ± SD for n = 3. ^∗^*p* < 0.05, ^∗∗∗^*p* < 0.001.

**Figure S10.** Cytotoxicity of Cel, Cel@M2-MSN, or Cel@M2-L-MSN at various drug concentrations (50-800 ng/mL) in RAW264.7 cells. Data were presented as mean ± SD for n = 3. ^∗^*p* < 0.05, ^∗∗∗^*p* < 0.001, ^∗∗∗∗^*p* < 0.0001.

**Figure S11.** The hemocompatibility of RBC (red blood cell) of (A) MSN, (B) Cel@MSN, (C) M2-L-MSN, and (D) Cel@M2-L-MSN at different nanoparticle concentrations. Distilled water and PBS (pH = 7.4, 10 mM) were used as the negative (-) and positive (+) controls, respectively. Data were presented as mean ± SD for n = 3.

**Figure S12.** (A) Images of spleens from different groups and (B) the corresponding spleen index. Data were presented as mean ± SD for n = 6. ^∗∗^*p* < 0.01, ^∗∗∗∗^*p* < 0.0001.

**Figure S13.** (A) Trabecular bone spacing (Tb.Sp), (B) trabecular thickness (Tb.Th), (C) tissue mineral density (TMD), (D) structure model index (SMI), and (E) total porosity of rats from different groups. Data were presented as mean ± SD for n = 6, ^∗^*p*< 0.05, ^∗∗^*p*< 0.01, ^∗∗∗^*p*< 0.001, ^∗∗∗∗^*p* < 0.0001.

**Figure S14.** The levels of blood **(**A) ALT, (B) AST, (C) BUN, and (D) CRE from different groups. Data were presented as mean ± SD for n = 6: ^∗^*p* < 0.05, ^∗∗^*p* < 0.01.

**Figure S15.** The (A) heart, (B) liver, (C) lung, and (D) kidney index of healthy or AIA rats from different groups. Data were presented as mean ± SD for n = 6.

**Figure S16.** Representative H&E staining images of heart, liver, Spleen, lung, and kidney tissue sections from different treatment groups. Scale bar = 100 μm.
